# State-of-Charge Estimation of Medium- and High-Voltage Batteries Using LSTM Neural Networks Optimized with Genetic Algorithms

**DOI:** 10.3390/s25154632

**Published:** 2025-07-26

**Authors:** Romel Carrera, Leonidas Quiroz, Cesar Guevara, Patricia Acosta-Vargas

**Affiliations:** 1 Universidad de las Fuerzas Armadas ESPE, Departamento de Ciencias de la Energía y Mecánica Sede Latacunga, Av. General Rumiñahui S/N, Sangolquí 171103, Ecuador; rdcarrera@espe.edu.ec (R.C.); laquiroz@espe.edu.ec (L.Q.); 2 Quantitative Methods Department, CUNEF Universidad, 28040 Madrid, Spain; cesar.guevara@cunef.edu; 3 Intelligent and Interactive Systems Laboratory, Universidad de Las Américas, Quito 170125, Ecuador

**Keywords:** monitoring system, genetic algorithms, SOC estimation, vehicle range

## Abstract

This study presents a hybrid method for state-of-charge (SOC) estimation of lithium-ion batteries using LSTM neural networks optimized with genetic algorithms (GA), combined with Coulomb Counting (CC) as an initial estimator. Experimental tests were conducted using medium-voltage (48–72 V) lithium-ion battery packs under standardized driving cycles (NEDC and WLTP). The proposed method enhances prediction accuracy under dynamic conditions by recalibrating the LSTM output with CC estimates through a dynamic fusion parameter α. The novelty of this approach lies in the integration of machine learning and physical modeling, optimized via evolutionary algorithms, to address limitations of standalone methods in real-time applications. The hybrid model achieved a mean absolute error (MAE) of 0.181%, outperforming conventional estimation strategies. These findings contribute to more reliable battery management systems (BMS) for electric vehicles and second-life applications.

## 1. Introduction

The increasing adoption of electric vehicles (EVs) and stationary energy storage systems has intensified research on accurate and efficient estimation of the state of charge of lithium-ion batteries, which is essential for battery management systems to ensure safety, performance, and longevity. SOC estimation is a complex problem due to the nonlinear dynamics of battery behavior under varying environmental and load conditions.

Accurate estimation of the state of charge in medium- and high-voltage batteries is essential to ensure the performance and safety of electric vehicles. However, the nonlinear and dynamic characteristics of batteries make this task particularly challenging under varying operating conditions, as noted by Plett et al. [1]. A first group of studies adopts model-based approaches that rely on physical representations of battery dynamics, primarily using Kalman filters and equivalent circuit models (ECMs). In the study by Manríquez et al. [2], long short-term memory (LSTM) neural networks were proposed to model these temporal complexities due to their ability to capture long-term dependencies in sequential data. Furthermore, optimizing LSTM models using genetic algorithms (GA) has been shown to improve estimation accuracy.

The study presented by Lee et al. [3] focuses on the estimation of the SOC by combining Gaussian processes with Bayesian filters. Two methods are proposed: a Gaussian process-assisted unscented Kalman filter (GP-UKF) and a Gaussian process-assisted particle filter (GP-PF). In both approaches, the Gaussian process regression (GPR) is trained to predict the battery voltage and dynamically update the covariance matrices of the respective filters. Using driving cycle data from UDDS and HWFET, the authors demonstrate significant improvements: the GP-UKF reduced the estimation error by up to 60% and uncertainty by approximately 80% compared to a classical UKF, while the GP-PF increased estimation accuracy by 58% and reduced uncertainty by 97% relative to a standard particle filter.

The study conducted by Shi et al. [4] use simulated data based on a lithium-ion battery equivalent circuit model featuring a second-order RC network. The proposed methodology includes parameter identification via a bias-compensated recursive least squares algorithm with adaptive forgetting factor, and the development of a novel estimator: the Improved Adaptive Square-Root Cubature Kalman Filter (IASRCKF). This estimator dynamically adjusts the error statistics window length to mitigate the impact of noise. The results demonstrate outstanding performance, with RMSE and MAE values of 0.18% and 0.15%, respectively—representing improvements of 40% and 44.4% over the standard ASRCKF approach. Additionally, robustness analyses under varying initial SOC values, current and voltage offsets, and different measurement noise covariances confirm the accuracy, robustness, and computational efficiency of the proposed method.

A second group of studies employs data-driven approaches based on conventional machine learning techniques, including multiple linear regression, decision trees, and random forests, often trained on real-time or historical battery data.

A study by Chandran et al. [5] employs machine learning algorithms to analyze and estimate the SOC, optimizing performance parameters through error analysis. The results indicate that artificial neural networks (ANN) and Gaussian process regression (GPR) are the most effective techniques, with the ANN achieving an MSE of 0.0004 and an RMSE of 0.00170, and the GPR yielding an MSE of 0.023 and an RMSE of 0.04118. These results highlight the superior accuracy of ANN and GPR in SoC prediction tasks.

In a related study, Amin et al. [6] tackled the challenge of estimating the range of electric motorcycles, a task complicated by the limited technical information provided by manufacturers. They developed an application that collects data on user behavior, weather, road conditions, and vehicle performance history. Several cloud-based machine learning algorithms were tested, with the support vector machine (SVM) model achieving the best results—yielding a mean absolute error of 150 m, which improved to 130 m after system optimization.

A study by Jain et al. [7] addresses the need for accurate prediction of SOC and driving range to reduce range anxiety in electric vehicles. Real-world trip data collected from electric scooters in India were used to train machine learning models, including k-nearest neighbors (KNN) and ensemble learning methods, combined with stochastic segmentation and Bayesian optimization. The best-performing models achieved prediction accuracies of 99.71% on the first validation trip and 98.04% on the second.

Pranav et al. [8] proposed a comparative machine learning algorithm for SOC estimation in electric vehicles, evaluating various approaches and highlighting the effectiveness of GPR. This study, published in Scientific Reports, trained models including SVM, neural networks, ensemble methods, and GPR using extensive real-world driving data collected from diverse drivers and conditions. Through statistical metrics applied to this field data, the study evaluated the SOC prediction accuracy, demonstrating that GPR achieved the highest accuracy with the lowest prediction errors, outperforming all other tested techniques. The authors concluded that GPR offers a reliable framework capable of modeling the complex relationship between real-time driving data and SOC, thereby improving SOC monitoring in electric vehicles under variable operating conditions.

A study presented by Ali et al. [9] focuses on battery-powered wireless sensor networks (WSNs) and the implementation of GPR in low-resource embedded systems. The proposed method is an adaptive SOC estimation approach using GPR trained with laboratory data under varying temperature conditions (5 °C to 45 °C) for three types of batteries (lithium-ion, NiMH, and Li-Poly). The GPR model employs an RBF kernel tuned via hyperparameter optimization, using voltage, capacity, and temperature as input features. Compared to other methods (e.g., polynomial regression and SVM) under the same conditions, the GPR model achieved mean absolute errors of approximately 2.0–2.5% and an RMSE of 0.30 in SOC prediction. Furthermore, the study demonstrates the feasibility of deploying this model on an ARM Cortex-M4 microcontroller, enabling real-time SOC estimation in WSN nodes. These results highlight the efficiency and lightweight nature of GPR for SOC estimation in IoT devices with constrained resources.

A third group of works leverages deep learning architectures such as LSTM, ANN, and CNN to capture temporal and nonlinear battery behaviors. LSTM networks have demonstrated strong potential for modeling the nonlinear behavior of battery systems. For instance, Bouktif et al. [10] developed an LSTM-based model optimized with genetic algorithms to identify time lags and optimal layer configurations. Several baseline machine learning models were evaluated, and the best features were selected using both filter-based and wrapper-based methods. Using electricity consumption data from the metropolitan area of France, the LSTM model achieved high accuracy and stability, with consistently low mean absolute error (MAE) and root mean square error (RMSE). The model achieved an RMSE of 0.61% for short-term forecasts and an average of 0.56% for medium-term forecasts, demonstrating that recurrent neural networks—specifically LSTM—are efficient and reliable tools for managing electric demand forecasting in smart grids.

A study by George et al. [11] addresses range anxiety in electric vehicles by implementing an electric vehicle model in MATLAB/Simulink and applying deep learning techniques to develop a neural network-based range prediction system. Their results showed that driving style, environmental conditions, battery parameters, and auxiliary loads significantly affect the vehicle’s range. Among the models tested, the bidirectional LSTM (BiLSTM) network achieved the lowest prediction error, with an RMSE of 0.029 km.

In a work by Oka et al. [12], an LSTM-based battery emulator was developed to predict the charging and discharging behavior of lithium-ion batteries (LIBs). The model was trained using both simulated data (via Dualfoil) and experimental data to forecast voltage profiles under galvanostatic conditions. The emulator demonstrated high accuracy, achieving coefficients of determination ($R^{2}$) of 0.98 with simulation data and 0.97 with experimental data.

Tian et al. [13] proposed a deep neural network (DNN) model for SOC estimation using voltage and charging current data sampled every 10 min. To enhance robustness against noise and sporadic errors, the approach was integrated with a Kalman filter. The methodology achieved SOC estimation errors below 2.03%, and reduced the mean squared error (MSE) to 0.385%, even under significant disturbances. Moreover, the model was adapted through transfer learning for various battery types and aging conditions, resulting in estimation errors below 3.146% for aged batteries and 2.315% for other battery chemistries.

A study by Tiwary et al. [14] investigated the influence of driving style on electric vehicle range estimation. The model incorporated three primary variables—speed, acceleration/deceleration, and battery SOC—along with driving data from four specific routes. An LSTM neural network was employed to capture temporal dependencies in driver behavior, and the model was optimized using the Adam algorithm with dropout regularization to prevent overfitting. The network achieved high predictive accuracy, with errors below 1% across all evaluated routes.

Kumar et al. [15] addressed the importance of accurately and reliably estimating the SOC of lithium-ion batteries to improve their performance, lifespan, and safety. Using voltage, current, and temperature data—collected under controlled thermal conditions (0 °C and 10 °C)—they applied machine learning and deep learning techniques based on artificial neural networks. The models achieved mean absolute errors (MAE) ranging from 0.0030 to 0.0035 and mean squared errors (MSE) between 0.0043 and 0.0047.

In a work by Gupta et al. [16], an electric vehicle was modeled and simulated in MATLAB/Simulink R2024b under various driving cycles. The resulting data were used to train a neural network for SoC estimation, initially using the coulomb counting method. After optimization with 40 neurons, the neural network achieved estimation errors below 1% for the Worldwide Harmonized Light Vehicles Test Procedure (WLTP-3) and below 3% for the Los Angeles 1992 (LA92) driving cycle.

A distinct set of studies explores Gaussian process regression (GPR)-based methods, which model uncertainty and nonlinearities in SoC estimation, incorporating factors such as battery expansion characteristics and thermal variability.

A study conducted by Yi et al. [17] investigates a novel SOC estimation method by leveraging physical changes in the battery. The work analyzes the variation in thickness (mechanical expansion) of Li-ion cells during charge/discharge cycles and its correlation with SOC. Based on this phenomenon, a GPR model is proposed, which uses both cell expansion and voltage characteristics as input features to predict SOC. Experimental results reveal very high accuracy: the maximum error does not exceed 0.0076 (dimensionless), and the RMSE remains around 0.0018 under constant charge/discharge conditions across various current rates. This approach demonstrates how GPR can incorporate mechanical indicators (expansion) to enhance SOC estimation in next-generation battery technologies.

An article presented by Hossain et al. [18] explores SOC estimation using GPR with a focus on thermal variability. Recognizing that battery dynamics are highly temperature-dependent, the study trains a GPR model using experimental data across a wide range of thermal conditions, from −10 °C to 25 °C. The resulting GPR model is capable of accurately predicting SOC throughout this range, demonstrating a global RMSE below 0.02 and a maximum absolute error under 0.1 across all tested temperatures. These results confirm that the model effectively captures the influence of temperature on battery behavior. Compared to other approaches, GPR proved to be more resilient to temperature fluctuations, maintaining reliable SOC estimations where traditional methods would suffer from degraded accuracy. This contribution is critical for optimizing battery management in applications with significant thermal variations (e.g., cold starts or hot climates), thereby enhancing the safety and efficiency of the BMS.

A study conducted by Gok et al. [19] addresses the challenge of accurately estimating the SOC of lithium-ion batteries under dynamic conditions and sub-zero temperatures, where internal resistance and usable capacity vary significantly. Real-world data were collected, including discharge rates from 0.2C to 2C, ambient temperature profiles ranging from 25 °C to −10 °C, battery surface temperature at five points, and voltage measurements, replicating scenarios based on the NEDC driving cycle. Two machine learning techniques were applied: NN and GPR, aimed at predicting SOC under complex operating conditions. Experimental results showed determination coefficients between 0.98 and nearly 1.00 across all scenarios. NN achieved higher accuracy and lower computational time under simple conditions, whereas GPR outperformed in complex nonlinear situations. Additionally, a reduction of up to 18.51% in discharged capacity was observed when sub-zero temperatures were combined with high discharge rates, underscoring the relevance of advanced SOC estimation techniques for demanding real-world applications.

A study by Bhattacharya et al. [20] addresses the challenge of accurately estimating the SOC in lithium-ion batteries, overcoming the limitations of static, temperature-sensitive models trained solely on laboratory data. A diverse dataset encompassing eight global driving cycles was employed, enabling the capture of a wide range of real-world conditions in electric vehicles. The proposed methodology integrates a generative model based on a stochastic variational Gaussian process (SVGP) with a deep neural network featuring incremental learning capabilities, forming an adaptive dual-model online learning system. This approach allows for progressively improved SOC estimation as new data become available, effectively addressing thermal variability. Experimental results demonstrate a substantial accuracy improvement, with the mean squared error reduced from 9 to 4 and the coefficient of determination increased by 90%, confirming the robustness and adaptability of the system.

Finally, several comprehensive or hybrid studies integrate multiple methodologies, offering comparative evaluations or extending SOC and SOH estimation beyond lithium-ion chemistries, such as sodium-based batteries. For instance, in the study by Xiang et al. [21], experimental tests were conducted on commercial sodium-ion batteries (SIBs) using a third-order equivalent circuit model, which was optimized through a particle swarm optimization algorithm for accurate SoC estimation. Three model-based approaches were evaluated: the extended Kalman filter (EKF), the unscented Kalman filter (UKF), and the particle filter (PF). Among them, the UKF demonstrated the highest accuracy, with mean absolute error and mean squared error values below 1.2% and 1.6%, respectively.

Wu et al. [22] report a SOC estimation method that incorporates GPR within an adaptive unscented Kalman filter. In this approach, GPR is employed to model the nonlinear relationship between measured parameters (current, voltage, temperature) and the SOC, and its predictions are fed into the UKF to enhance online SOC estimation. In comparative tests, the GPR-based UKF demonstrated high prediction accuracy, maintaining SOC error below 1% under various loading conditions. The inclusion of GPR allowed the filter to adapt more effectively to system variations without the need to re-identify internal models, suggesting a promising framework for battery management systems where both precision and robustness are critical.

In this study, the term medium- and high-voltage lithium-ion batteries refers to battery systems with nominal voltages exceeding 60 V and up to 400 V, commonly found in electric motorcycles, hybrid electric vehicles (HEVs), and electric cars. These packs consist of multiple lithium-ion cells connected in series and/or parallel, resulting in higher energy storage capacity and power output compared to typical low-voltage commercial battery packs (e.g., 12–48 V). Unlike low-voltage lithium batteries used in consumer electronics or light electric vehicles, medium- and high-voltage batteries operate under more demanding thermal, electrical, and safety constraints. Therefore, SOC estimation for these systems requires robust, accurate, and scalable algorithms capable of handling rapid transients and voltage nonlinearities. The distinction is essential to contextualize the relevance and applicability of the proposed estimation approach under realistic high-voltage EV operating conditions.

### Research Gap, Problem Statement, and Objectives

While numerous studies report state-of-charge estimation accuracies exceeding 98%, such performance figures should be interpreted with caution. First, many of these works rely on highly controlled datasets that do not reflect the full variability of real-world driving scenarios, such as rapid temperature fluctuations or mixed urban/highway profiles. Second, dataset sizes are often relatively small (typically comprising only a few hundred cycles), which increases the risk of overfitting, particularly when complex models such as LSTM networks are employed. Third, methodological details are sometimes insufficiently reported, making it difficult to assess the true generalization capabilities of the proposed models.

Therefore, although these high reported accuracies demonstrate the potential of advanced machine learning techniques, they may overstate real-world performance under the practical constraints of battery management systems, where sensor noise, battery degradation, and hardware limitations play a significant role.

Consequently, a clear gap exists between the idealized, upper-bound performance typically presented in the literature and the practical accuracy achievable in an embedded BMS within an electric vehicle powertrain. The problem addressed in this work is twofold: The development of an SOC estimator that consistently maintains acceptable accuracy (above 90%) across dynamically varying load profiles, thermal drift, and cell ageing. Ensuring that the model’s computational and memory footprint fits within the constraints of typical embedded BMS hardware, without compromising prediction quality. To address this gap, the present study pursues the following objectives:

- Design and implement a hybrid LSTM + GA framework capable of automating the hyperparameter optimization process for SOC prediction;
- Compare the hybrid model’s performance in terms of prediction accuracy, convergence speed, and computational cost against two baselines: a conventional LSTM model and a multiple linear regression (MLR) estimator;
- Validate all models using a comprehensive dataset that includes urban and highway driving cycles, temperature fluctuations, and cell aging profiles—realistically representing the operational conditions of electric vehicles;
- Assess the feasibility of deploying the proposed estimator on embedded battery management system (BMS) platforms by evaluating memory usage, training and inference time, and expected energy consumption under fixed-point arithmetic implementation.

The present study develops and evaluates an adaptable monitoring system for medium- and high-voltage electric propulsion systems. This approach enables real-time measurement of key parameters, including the state of charge of the test vehicle’s battery. The system integrates machine learning techniques to provide accurate SOC estimations. Experimental tests were conducted in Latacunga, Ecuador, using standardized driving cycles such as the New European Driving Cycle (NEDC), the Worldwide Harmonized Light Vehicles Test Procedure (WLTP), and custom-designed routes. Data acquisition is carried out via sensors and visualized in real-time using a central microprocessor. Unlike previous research focused on electric vehicle batteries in four-wheeled vehicles operating on flat terrain, this study adapts its methodology to electric motorcycles operating in mountainous environments. It introduces the use of accelerator angle sensors and applies genetic algorithms to enhance energy management and battery reliability.

The remainder of the article is organized as follows: Section 2 details the materials and methods used for data collection, including standardized driving cycles. Section 3 describes the implementation of the proposed monitoring system, outlining the electronic signal flow from acquisition to visualization. Section 4 presents the experimental results and performance metrics of the evaluated models. Section 5 discusses and analyzes these results in comparison with related studies. Finally, Section 6 provides the conclusions and outlines potential improvements for future iterations of the prototype.

## 2. Materials and Methods

This section outlines the methodologies, techniques, and resources used to estimate the SOC in medium- and high-voltage batteries employed in electric propulsion systems for both two- and four-wheeled vehicles. Accurate SOC estimation is essential for preventing operational risks such as overcharging or deep discharging, which can degrade battery cells and reduce the vehicle’s driving range—a critical factor in the widespread adoption of electric mobility. In addition, electric propulsion systems are subject to operational challenges such as thermal variability, battery aging, and highly dynamic charge/discharge cycles. These conditions require adaptive models capable of integrating real-time data to ensure robust and reliable SOC estimation [23].

Prior to SOC estimation, the state of health of each cell must be determined by measuring its remaining capacity and internal resistance through controlled cycling tests. These SoH metrics are used to calibrate the SOC prediction model, as battery degradation introduces significant bias when estimates rely solely on voltage and current readings.

In this study, the EBC-A40L battery tester was used to assess the actual capacity and health of the LiFePO_4_ cells (3.2 V, 50 Ah). This equipment enables controlled charge–discharge cycles under standardized conditions (ambient temperature: 25 ± 2 °C; discharge rate: 0.5C). The cell is first fully charged to 3.65 V using a constant current–constant voltage (CC–CV) profile and then discharged to 2.5 V while recording the discharged capacity.

The measured capacity is compared to the nominal value to calculate the SoH using Equation (1).

(1) $$\mathrm{SoH}\;\left(\%\right)=\left(\frac{\mathrm{Measured}\;\mathrm{Capacity}}{\mathrm{Initial}\;\mathrm{Capacity}}\right)\times 100$$

In addition, the EBC-A40L monitors critical parameters such as the voltage curve during discharge, internal resistance, and thermal stability, enabling the identification of accelerated degradation or electrochemical inhomogeneities. This method ensures accurate and reproducible estimation of SoH, which is essential for predicting remaining battery life and validating the reliability of SOC estimation models in real-world automotive applications.

### 2.1. LSTM Neural Networks

A long short-term memory (LSTM) neural network is a type of recurrent neural network (RNN) specifically designed to process sequential data and capture long-term dependencies. Unlike traditional RNNs, LSTMs incorporate memory cells along with gate mechanisms—namely the input, output, and forget gates—that allow the model to selectively retain or discard information over time. This architectural design enables LSTMs to effectively model complex temporal dynamics and mitigate issues such as vanishing gradients. As a result, they are particularly well-suited for tasks involving time-series forecasting, sequential sensor data, and natural language processing, where learning from long-term context is essential [24].

The forget gate determines which information in long-term memory should be discarded. Equation (2) uses a sigmoid function to produce a value between 0 and 1, indicating the degree of forgetting. The weight $W_{f}$ adjusts the importance of the previous $h_{t-1}$ and current inputs $x_{t}$, while the bias $b_{f}$ compensates for possible deviations.

(2) $$f_{t}=\sigma \left(W_{f}\;\cdot \;\left[h_{t-1},x_{t}\right]+b_{f}\right)$$

The input gate (Equation (3)) regulates the new information incorporated into the memory. It uses a sigmoid function that evaluates the inputs, weights $W_{i}$, and bias $b_{i}$. Its output, which ranges from 0 to 1, indicates the proportion of relevant data that should be added to the cell state.

(3) $$i_{t}=\sigma \left(W_{i}\left[h_{t-1},x_{t}\right]+b_{i}\right)$$

Equation (4) generates a proposal of new information for the cell state. It uses the hyperbolic tangent function to transform the inputs $h_{t-1}$$x_{t}$ with weights $W_{c}$ and bias $b_{c}$. Its output is a filtered representation between −1 and 1 of the incoming data, which is used if the input gate allows:

(4) $${\tilde{C}}_{t}=\tanh\left(W_{c}\left[h_{t-1},x_{t}\right]+b_{c}\right)$$

The cell state (Equation (5)) combines the retained information with the new data. This operation multiplies the previous state $C_{t-1}$ by the forget gate output $f_{t}$ and adds the candidate content ${\tilde{C}}_{t}$ weighted by the input gate $i_{t}$. The result is an updated memory that reflects both experience and new operating conditions:

(5) $$C_{t}=f_{t}\;{\bar{C}}_{t-1}+i_{t}\;{\bar{C}}_{t}$$

Equation (6) defines the gate that determines how much information from the cell state should be transmitted to the next time step or the output layer. Its output $o_{t}$ modulates the influence of the current state $C_{t}$ on the final result:

(6) $$o_{t}=\sigma \left(W_{o}\left[h_{t-1},x_{t}\right]+b_{o}\right)$$

The hidden state (Equation (7)) represents the intermediate output of the LSTM node. It is obtained by multiplying the output gate output $o_{t}$ by the hyperbolic tangent of the cell state $C_{t}$. This combination enables the network to retain relevant information while attenuating extreme values:

(7) $$h_{t}=o_{t}\;\tanh\left(C_{t}\right)$$

Equation (8) for the predictive output transforms the hidden state $h_{t}$ into an estimate. It uses an activation function—such as sigmoid—to scale values within the required bounds. The weights *W* and bias *b* define how each feature influences the final prediction:

(8) $$\mathrm{Predictive}\;\mathrm{data}=\mathrm{Activation}(W\;\cdot \;h_{t}+b)$$

Optimization algorithms, such as Adam or root mean square propagation (RMSProp), iteratively adjust the LSTM weights to reduce the mean squared error.

#### Multiscale Feature Alignment and SoH Decoupling

To address the coexistence of variables with different temporal dynamics, a two-stage preprocessing and feature alignment approach was implemented.

First, high-frequency signals such as voltage, current, acceleration, and throttle angle were collected at a rate of 2.6 Hz (every ∼0.385 s). In contrast, the state of health (SoH) was considered a slowly varying parameter, typically changing over much longer periods (daily or hourly). To integrate this disparity,

- SoH values were interpolated to remain constant over fixed one-minute intervals (pseudo-static behavior),
- A dual-input architecture was evaluated, where SoH acted as a contextual feature concatenated only at the input gate of the LSTM and not recurrently propagated.

Second, all inputs were normalized individually using a time-aware z-score method, ensuring that variables with different magnitudes and sampling rates did not dominate the training loss.

This design ensures the robustness of the LSTM against temporal mismatches and avoids overfitting to spurious short-term correlations with slow-changing parameters.

### 2.2. Genetic Algorithms

Genetic algorithms (GAs) are optimization techniques inspired by the principles of biological evolution. They employ mechanisms such as selection, crossover, and mutation to search for optimal or near-optimal solutions to complex problems. When applied to the optimization of LSTM neural networks, GAs aim to enhance model performance by tuning hyperparameters—such as the number of layers, the number of neurons per layer, and the learning rate—or, in some cases, by optimizing the network’s weights [25]. Through iterative evaluation of candidate configurations and selective reproduction of the best-performing solutions, GAs efficiently explore a vast and nonlinear search space. This approach improves the predictive capabilities of LSTM networks, particularly in applications involving time-series forecasting and other sequential data modeling tasks [26].

GAs employ various mathematical operators to optimize LSTM networks. The fitness function (Equation (9)) evaluates how good a solution (i.e., an individual) is for the problem at hand. In this context, $y_{i}$ it represents the actual value, ${\hat{y}}_{i}$ denotes the value predicted by the network, and *n* is the number of samples. Typically, in optimizing LSTM networks, the fitness function is based on the prediction error, such as the mean squared error.

(9) $$\mathrm{Fitness}\left(x\right)=\frac{1}{n}\sum_{i=1}^{n}{\left(y_{i}-{\hat{y}}_{i}\right)}^{2}$$

Equation (10) selects the best solutions to generate the next generation. A common method is the roulette wheel selection. P(i) is the selection probability of each individual, $f_{i}$ is the fitness value of the individual, and *N* is the population size:

(10) $$P\left(i\right)=\frac{f_{i}}{\sum_{j=1}^{N}f_{j}}$$

Through Equation (11), the parameters of two individuals (parents) are combined to produce new offspring. α is a random coefficient between 0 and 1. Uniform crossover is often used:

(11) $${\mathrm{Son}}_{1}=\alpha \;\cdot \;{\mathrm{Father}}_{1}+\left(1-\alpha \right)\;{\mathrm{Father}}_{2}$$

In Equation (12), some parameters of an individual are randomly changed to explore new solutions. δ is a random value within a predefined range:

(12) $${\mathrm{Gen}}_{i}={\mathrm{Gen}}_{i}+\mathrm{range}\;\;\cdot \;\delta$$

The population is replaced by a combination of the best individuals (elitism) and the new offspring generated by crossover and mutation. In Equation (13), the algorithm stops when a stopping criterion (ϵ is a small threshold) is met, such as reaching a maximum number of iterations or achieving a minimal improvement in the fitness function [27].

(13) $$\left|{\mathrm{Fitness}}_{\mathrm{actual}}-{\mathrm{Fitness}}_{\mathrm{previous}}\right|<\epsilon$$

### 2.3. Mathematical Formalization of LSTM Network Optimization Using Genetic Algorithms

To optimize the LSTM network architecture, a genetic algorithm was implemented to automatically select key hyperparameters, including the number of hidden units, learning rate, batch size, and dropout rate. The following GA parameters were used: a population size of 30 individuals, a maximum of 50 generations, a crossover probability of 0.8, and a mutation probability of 0.1. These values were chosen based on preliminary sensitivity analysis and aligned with recommendations from related works (Bouktif et al. [10]; Kumar et al. [15]).

A smaller population (<20) resulted in premature convergence and limited diversity, while larger populations (>50) significantly increased computation time without substantial accuracy gain. The number of generations (50) provided a trade-off between exploration and convergence stability. The crossover rate (0.8) ensures effective combination of candidate solutions, while the mutation rate (0.1) introduces sufficient variability to avoid local minima. A binary encoding scheme was used to represent the candidate solutions, where each chromosome encodes a unique combination of LSTM hyperparameters. The fitness function was defined as the inverse of the RMSE on the validation dataset, ensuring that the GA prioritizes configurations that yield better SOC estimation accuracy.

**Figure 1:**
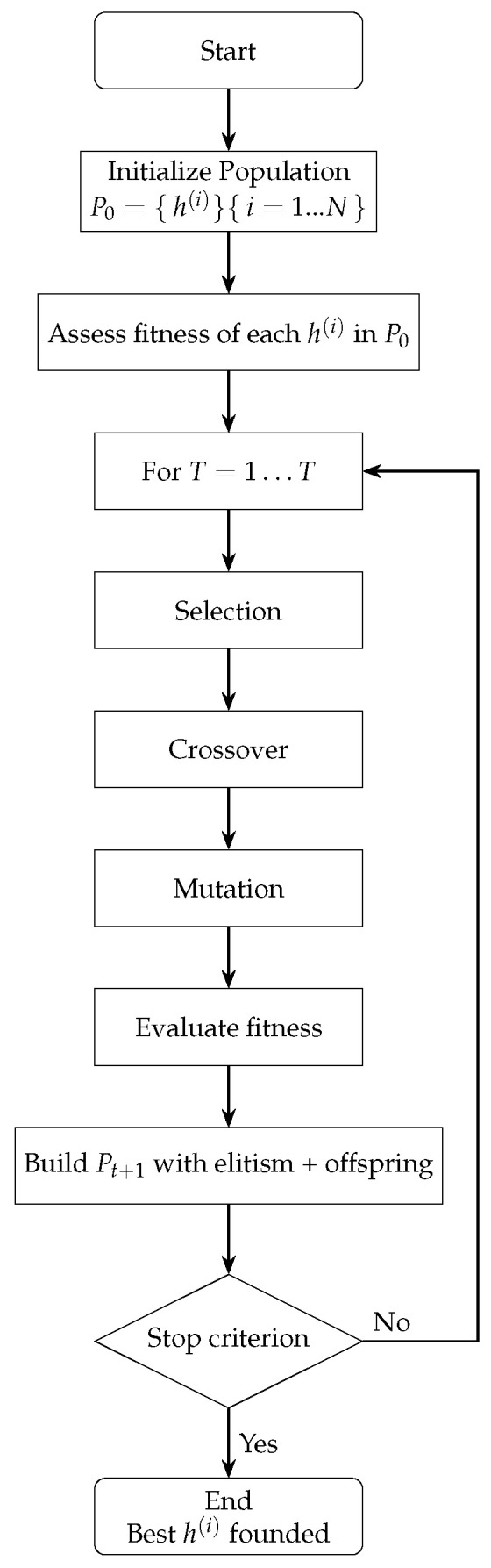
LSTM neural network parameter optimization flowchart.

As shown in Figure 1, the optimization process begins with the generation of an initial population of hyperparameter vectors for the LSTM model. Each vector is used to train the network, and its fitness is evaluated on a validation dataset. In each generation, parent vectors are selected based on a probability proportional to their fitness. These selected individuals are then combined using crossover operations and undergo random mutations to produce offspring. The fitness of the offspring is re-evaluated, and a new population is formed by preserving an elite subset of the best-performing individuals and complementing them with the newly generated offspring. This evolutionary cycle—comprising selection, crossover, mutation, and replacement—continues until a predefined number of generations is reached or no further improvement in fitness is observed. The hyperparameter vector with the highest fitness score is then selected as the optimal configuration for the LSTM network [28].

#### 2.3.1. Hyperparameter Vector

The hyperparameter vector (Equation (14)) in LSTM networks optimized with genetic algorithms groups key parameters into three categories:

1. Architectural hyperparameters (e.g., number of LSTM layers $h_{1}$, neurons per layer $h_{2}$, and auxiliary components such as dropout rate $h_{3}$ or dense layer size $h_{4}$),
2. Training hyperparameters (e.g., learning rate $h_{5}$, batch size $h_{6}$, and sequence length $h_{7}$, which defines the input time window), and
3. Optimization-specific parameters (e.g., mutation rate $h_{8}$ and crossover strategy $h_{9}$).

The latter is critical for guiding the exploration and exploitation of the genetic algorithm within the search space. This vector determines the model’s capacity, its convergence during training, and its adaptability to specific tasks, such as time series forecasting [29].

(14) $$h=(h_{1},h_{2},\ldots ,h_{d})$$

#### 2.3.2. Evaluation Function (Fitness)

For each individual *h*, we train the LSTM using the corresponding hyperparameters and evaluate the MSE on a validation set. The fitness function is defined by Equation (15), where the negative sign is used to convert the minimization of the MSE into a maximization of the fitness [30].

(15) $$F\left(h\right)=-{\mathrm{MSE}}_{\mathrm{val}}\left(\mathrm{LSTM}\left(h\right)\right)$$

#### 2.3.3. Roulette Wheel Selection

In genetic algorithms, this method is used to select individuals from a population ${\left\{h^{\left(i\right)}\right\}}_{i=1}^{N}$ according to their fitness *F*, by assigning selection probabilities proportional to their performance. In Equation (16), $F(h^{\left(i\right)})$ represents the fitness function of individual $h^{\left(i\right)}$, and $\sum_{j=1}^{N}F\left(h^{\left(j\right)}\right)$ is the total fitness of the population. $P_{i}$ denotes the probability of selecting the *i*-th individual. This mechanism favors individuals with higher fitness, replicating the “survival of the fittest” principle to generate the next generation of candidate solutions [31].

(16) $$P_{i}=\frac{F(h^{\left(i\right)})}{\sum_{j=1}^{N}F\left(h^{\left(j\right)}\right)}$$

#### 2.3.4. Uniform Crossover

Uniform crossover is a genetic operator used in evolutionary algorithms to combine two parent vectors, $h^{\left(p1\right)}$ and $h^{\left(p2\right)}$, by generating a child whose genes are randomly selected from each parent. In Equation (17), for each *k*-th gene of the child $h_{k}^{\left(\mathrm{child}\right)}$, a value $u_{k}$ is sampled from a uniform distribution U(0,1): if $u_{k}<0.5$, the gene is inherited from the first parent, $h_{k}^{\left(p1\right)}$; otherwise, from the second parent, $h_{k}^{\left(p2\right)}$. This method ensures a random and independent mixing of hyperparameters, promoting diversity in the population by avoiding bias toward specific segments of the parents, thereby facilitating a balanced exploration of the search space [32].

(17) $$h_{k}^{\left(\mathrm{child}\right)}=\left\{\begin{array}{cc} h_{k}^{\left(p1\right)}, & \mathrm{if}\;u_{k}<0.5,\\ h_{k}^{\left(p2\right)}, & \mathrm{if}\;u_{k}\geq 0.5 \end{array}\right.$$

#### 2.3.5. Gaussian Mutation

Gaussian mutation is an operator in genetic algorithms that introduces variability in an individual’s genes through random perturbations based on a normal distribution. For each gene $h_{k}$, with mutation probability $p_{\mathrm{mut}}$, Equation (18) is applied, where $h_{k}$ represents the original value of the *k*-th gene, and $h_{k}^{\prime }$ is the mutated gene value after perturbation. σ denotes the mutation amplitude, which controls the standard deviation of the noise (i.e., the magnitude of the variation). N(0,1) is a standard normal random variable (mean 0, standard deviation 1) that generates random fluctuations around the original value. This method allows controlled exploration of the search space, favoring small adjustments (due to the high probability density of N(0,1) near zero) while maintaining genetic diversity. The mutation probability $p_{\mathrm{mut}}$ regulates the frequency of mutations, balancing exploration and exploitation [33].

(18) $$h_{k}^{\prime }=h_{k}+\sigma \;N\left(0,1\right)$$

#### 2.3.6. Population Update

Population update in genetic algorithms defines how the new generation ($P_{t+1}$) is formed by combining the best individuals from the current population ($P_{t}$) with the newly generated offspring. According to Equation (19), *E* represents the number of elite individuals (i.e., the best solutions from $P_{t}$), preserved through elitism to ensure that optimal solutions are not lost. *N* denotes the total population size, where N−E corresponds to the offspring created using genetic operators (crossover, mutation). The set $\mathrm{top}\text{-}E(P_{t})$ refers to the *E* individuals with the highest fitness in $P_{t}$, and offspring represents the set of N−E new individuals generated through parent selection and recombination. This strategy balances the exploitation of existing solutions (elitism) with the exploration of new ones (offspring), ensuring convergence toward optimal solutions without compromising genetic diversity [34].

(19) $$P_{t+1}=\left\{\mathrm{top}\text{-}E\left(P_{t}\right)\right\}\cup \left\{\mathrm{offspring}\right\}$$

#### 2.3.7. Stopping Criterion

The stopping criterion in genetic algorithms determines when to terminate the evolutionary process based on two conditions:

1. Reaching a maximum number of generations $T_{T}$, a predefined parameter that limits the execution to avoid excessive computational costs; or
2. Absence of improvement in the best fitness value of the population, $\max_{i}F\left(h^{\left(i\right)}\right)$, where $F(h^{\left(i\right)})$ is the fitness function of individual $h^{\left(i\right)}$ over *G* consecutive generations, indicating algorithm convergence.

Here, *G* acts as a patience threshold: if there is no increase in the maximum fitness after *G* iterations, it is assumed that no better solutions will be found. This mechanism balances search space exploration and computational efficiency, avoiding redundant executions [35].

### 2.4. Multiple Linear Regression

The multiple linear regression model assumes that the dependent variable can be expressed as a linear combination of the independent variables plus an error term. It is defined by Equation (20), where $a_{0}$ is the constant term, $a_{1},a_{2},\ldots ,a_{n}$ are the coefficients that determine the influence of each variable $x_{n}$, and ε is the error or residual, assumed to follow a normal distribution [36]:

(20) $$y=a_{0}+a_{1}x_{1}+a_{2}x_{2}+\ldots +a_{n}x_{n}+\varepsilon$$

To determine the values of $a_{1},a_{2},\ldots ,a_{n}$, the mean squared error (MSE) is minimized over the training dataset using Equation (21). The cost function to be minimized is the following:

(21) $$\mathrm{MSE}=\frac{1}{N}\sum_{i=1}^{N}{\left(y_{i}-a_{0}-\sum_{j=1}^{n}a_{j}x_{ij}\right)}^{2}$$

The analytical solution is obtained by solving the normal equations (Equation (22)). *X* is the design matrix (each row is a sample and each column is a variable, including a column of ones for the intercept), and y is the response vector:

(22) $$\hat{a}={\left(X^{T}X\right)}^{-1}X^{T}y$$

In the Python (3.12.11) programming environment, the scikit-learn library handles this calculation using the fit() method of the LinearRegression object. As a data preprocessing step before training the model, the features are normalized using StandardScaler. This involves transforming each variable *x* according to Equation (23). Here, μ is the mean and σ the standard deviation of the variable. This transformation ensures that all variables share a similar scale, improving the numerical stability of the training process:

(23) $$x_{\mathrm{scaled}}=\frac{x-\mu }{\sigma }$$

The train_test_split function splits the dataset into a training set (used to fit the model) and a test set (used to evaluate the model’s predictive capability). During training and validation, the model’s coefficients are calculated by minimizing the sum of squared errors on the training set.

Once the model is trained, it makes predictions $\hat{y}$ on the test set (Equation (24)):

(24) $$\hat{y}=a_{0}+a_{1}x_{1}+a_{2}x_{2}+\ldots +a_{p}x_{p}$$

The model’s performance is then evaluated using various metrics that quantify how far, on average, the predictions of the model are from the actual values [37].

Mean squared error (MSE): Equation (25) is the average of the squared errors between the actual and predicted values, heavily penalizing significant errors.

(25) $$\mathrm{MSE}=\frac{1}{N}\sum_{i=1}^{N}{\left(y_{i}-{\hat{y}}_{i}\right)}^{2}$$

Root mean squared error (RMSE): Equation (26) is the square root of the MSE, allowing the error to be interpreted in the same units as the original variable.

(26) $$\mathrm{RMSE}=\sqrt{\mathrm{MSE}}$$

Mean absolute error (MAE): Equation (27) is the average of the absolute differences between the actual and predicted values, providing a direct measure of the mean error.

(27) $$\mathrm{MAE}=\frac{1}{N}\sum_{i=1}^{N}\left|y_{i}-{\hat{y}}_{i}\right|$$

### 2.5. Materials

In this study, a real-time monitoring system is employed, equipped with sensors for temperature (°C), current (A), voltage (V), acceleration (m/s^2^), and accelerator angle (°), as well as an integrated visualization interface for displaying data during testing. The information from these independent variables is recorded to enable accurate estimation of the state of charge.

Standardized driving cycles—including the New European Driving Cycle (NEDC) and the Worldwide Harmonized Light Vehicle Test Procedure (WLTP)—along with custom routes (e.g., inter-campus travel scenarios), are used to capture realistic operating conditions. The resulting SOC data are stored in a time-series format to build a historical dataset, which is then used to train both the LSTM neural network and the multiple linear regression model.

This dataset integrates data collected under various driving conditions, supporting effective hyperparameter optimization. The computational infrastructure processes this information by leveraging the temporal prediction capabilities of the LSTM network in combination with the interpretability and precision of the MLR model, with the goal of developing a robust hybrid estimator for SOC prediction.

There are various approaches for estimating the state of charge in electric vehicle batteries. The open-circuit voltage (OCV) method is based on the empirical relationship between SOC and resting voltage. It provides good accuracy under steady-state conditions but requires long pauses for measurement. Coulomb counting integrates the charge/discharge current and is simple and fast in real time, but it accumulates errors due to drift and capacity variations. Equivalent circuit models (RC networks with Kalman filters) incorporate electrical dynamics and compensate for sensor noise, improving stability under varying conditions. Artificial intelligence methods (neural networks, LSTM, SVM) learn nonlinear patterns and capture thermal and aging effects, although they require large amounts of data and computational power for training. Finally, hybrid approaches that combine two or more techniques (Coulomb counting and LSTM) integrate the speed and simplicity of some methods with the error correction and adaptability of others, achieving the best trade-off between accuracy, robustness to dynamic changes, and implementation cost.

In this study, a hybrid method is used to calculate SOC that integrates two complementary approaches. On one hand, Coulomb counting calculates the variation in SOC based on the integration of the actual current supplied to or drawn from the battery, adjusted by a Coulombic efficiency factor. On the other hand, an LSTM model is trained on time series of current, voltage, temperature, acceleration, throttle angle, and battery state-of-health data, enabling it to capture nonlinearities and thermal dynamics of the system under real operating conditions.

As shown in Equation (28), at each time instant *t*, the values of $SOC_{CC}\left(t\right)$ and $SOC_{LSTM}\left(t\right)$ are obtained and combined through a weighting factor α, either fixed or adaptively chosen between 0.5 and 0.9. This fusion leverages the short-term accuracy and speed of Coulomb counting with the LSTM’s capacity to correct accumulated errors and respond to dynamic events such as accelerations or thermal changes. The result is a more accurate and robust SOC estimate throughout the entire operating cycle.

(28) $$SOC_{\mathrm{hybrid}}\left(t\right)=\alpha \;SOC_{CC}\left(t\right)+\left(1-\alpha \right)\;SOC_{LSTM}\left(t\right)$$

In Equation (28), the parameter α∈[0,1] governs the contribution of each estimator in the fusion process:

The selection of α was based on a cross-validation strategy using empirical data from five driving cycles. To improve estimation accuracy and account for varying reliability of each estimator under different load conditions, α was adjusted dynamically according to current intensity and LSTM stability, as shown in Table 1.

This adaptive strategy allows the model to rely more on CC in low-current regions where drift is minimal, use a balanced fusion in moderate regimes, favor LSTM in high-transient regions where CC becomes less accurate due to integration drift. The thresholds were derived empirically to minimize RMSE over a validation dataset. Future work may explore reinforcement learning or Kalman-based dynamic weighting.

The dataset used in this study is derived from extensive experimental trials in which key parameters of an electric vehicle battery are continuously monitored. State of charge values are recorded at non-uniform time intervals, and each evaluated driving cycle is represented as an independent time series consisting of 5213 data instances for prediction purposes.

The dataset includes a dedicated SOC column that displays a nonlinear, monotonically decreasing trend, with gradually diminishing values that correspond to the battery’s discharge behavior. Relevant features for training the LSTM network include characteristic discharge patterns with fluctuations associated with variations in load, operating conditions, or user behavior. Raw sensor measurements are initially recorded with a precision of three decimal places. To reduce statistical noise near the limit of instrument resolution, all signals are preprocessed using a five-sample moving average filter. Subsequently, the data are quantized to two decimal places before model training. This procedure ensures that the LSTM network captures true battery dynamics rather than high-frequency fluctuations introduced by measurement noise.

**Figure 2:**
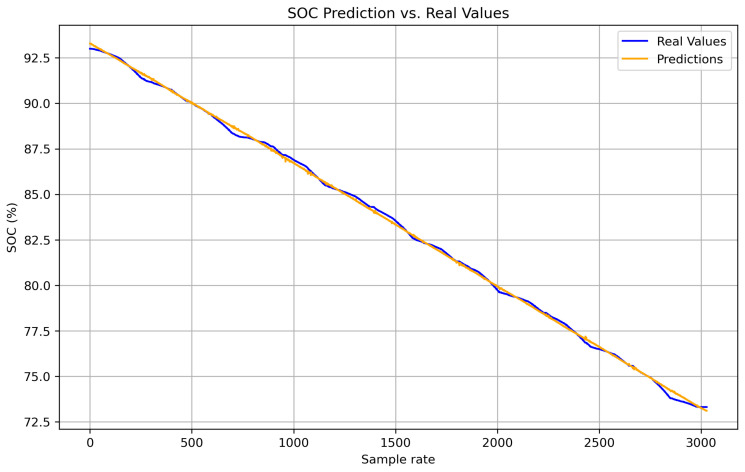
Battery state of charge during the test cycle.

Figure 2 shows the real SOC curve (blue line) and the SOC estimated by the hybrid method (orange line), based on Coulomb counting combined with LSTM. Both follow the same downward trend, confirming the estimator’s reliability. The hybrid method accurately reproduces the global discharge slope (from approximately 93% to 72%) due to the contribution of Coulomb counting, while the small oscillations captured reflect the LSTM’s ability to correct for dynamic variations (such as current, temperature, and acceleration). With an RMSE of 0.48% and an MAE of 0.36%, the model achieves an error below 1%, making it suitable for real-time energy management. The weighting factor α enables the model to combine long-term stability with the correction of short-term deviations.

In the analysis of SOC in electric vehicle batteries, autocorrelation plots are essential tools for examining how variables correlate over time. These plots enable the identification of temporal patterns, dependencies, or noise within the data—factors that are critical for the development of accurate SOC prediction models. Autocorrelation analysis reveals whether past values of a variable significantly influence its future values, thus providing key insights for optimizing battery management algorithms and improving the predictive performance of time-series models.

**Figure 3:**
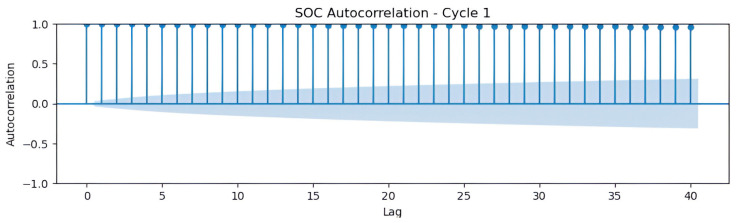
Autocorrelation of the state of charge in a test cycle.

In Figure 3, the autocorrelation function (ACF) graph of SOC in cycle 1 reveals a complex temporal structure, with significant autocorrelations at lags 0, 5, 15, 20, and 40. These findings highlight a slow decay, suggesting a trend, and recurring peaks that may indicate seasonal patterns. The unusual value of 1.0 at lag 40 might signal strong seasonality, requiring additional validation. These patterns justify the use of integrated autoregressive moving average (ARIMA) or seasonal ARIMA models, with autoregressive components (p=2 for lags 5 and 15) and seasonality (m=40), combined with differencing to ensure stationarity. It is advisable to analyze the time series in its temporal domain to rule out anomalies and perform stationarity tests—such as Dickey–Fuller—to optimize predictive modeling.

### 2.6. Data Preprocessing Justification

To ensure robust signal conditioning prior to model training, this study applied two preprocessing techniques: a moving average filter and numerical quantization. Their configurations were selected based on a quantitative analysis of their impact on signal fidelity and noise attenuation.

#### 2.6.1. Moving Average Filter

We tested window sizes of 3, 5, 7, and 9 samples on raw voltage, current, and acceleration signals. As summarized in Table 2, a five-sample window achieved the best trade-off between noise suppression and temporal accuracy. Specifically, this configuration yielded a low RMSE while preserving key dynamic transitions in the signals. For instance, the RMSE for the current signal increased from 1.20 A (window = 3) to 2.25 A (window = 9), indicating that larger windows overly smoothed transient behavior.

**Figure 4:**
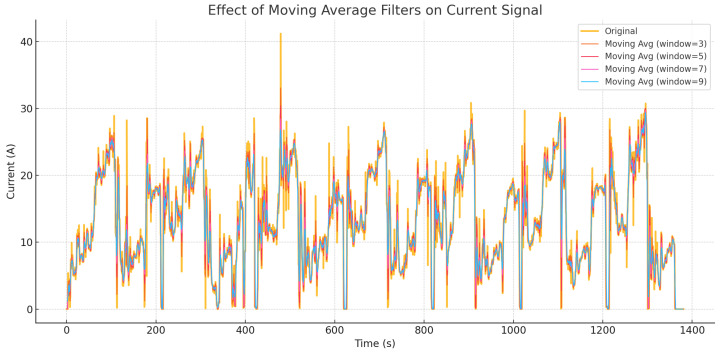
Effect of moving average filter window sizes (3, 5, 7, and 9) on the raw current signal. A 5-sample window achieves the best trade-off between smoothing and temporal fidelity.

A visual comparison (Figure 4) confirms that the five-sample window effectively suppresses sensor noise without significantly distorting sharp variations, such as acceleration bursts or regenerative braking events.

#### 2.6.2. Quantization Analysis

To reduce statistical noise near the sensor resolution limit, we evaluated the effect of rounding all signal values to 1, 2, and 3 decimal places (Table 3). We computed the signal-to-quantization-noise ratio (SQNR) for each case. Results showed that rounding to two decimals preserved high SQNR values (>54 dB for current) and introduced no measurable degradation in voltage or acceleration signals (SQNR → *∞*). In contrast, one-decimal quantization caused a significant SQNR drop, indicating information loss in sensitive variables.

Hence, quantization to two decimals was selected to ensure numerical stability during model training while retaining essential signal detail.

#### 2.6.3. Dataset Description and Statistical Summary

As shown in Table 4 the dataset used in this study comprises a total of 5213 time-series samples, collected from real-world testing under five distinct driving scenarios.

Each sample corresponds to a 0.385-s interval, resulting in a total coverage time of approximately 33.4 min.

Table 5 summarizes the main descriptive statistics of the variables used in model training:

### 2.7. OCV–SOC Curve Fitting

To model the relationship between open circuit voltage and state of charge, a 6th-degree polynomial fitting method was employed. Experimental OCV data were obtained under static relaxation conditions at an ambient temperature of 25 °C. After each discharge or charge step, the battery was allowed to rest until voltage stabilization (typically 1 h), and the corresponding SOC was recorded.

The resulting OCV–SOC dataset was fitted using the following polynomial expression (Equation (29)).

(29) $$\mathrm{OCV}\left(x\right)=a_{6}x^{6}+a_{5}x^{5}+a_{4}x^{4}+a_{3}x^{3}+a_{2}x^{2}+a_{1}x+a_{0}$$

where *x* is the SOC (0–1), and $a_{i}$ are the fitted coefficients derived via least squares regression.

This approach enables smooth interpolation across the entire SOC range and avoids the stepwise discontinuities associated with traditional lookup tables.

Temperature Consideration: The OCV–SoC model was developed under nominal conditions (25 °C), and the thermal effect on OCV was not modeled. Although this assumption holds in controlled environments, the influence of temperature on OCV may become significant at low temperatures. This limitation is acknowledged and will be addressed in future work using temperature-dependent OCV profiles.

## 3. Proposed Monitoring System

This section presents a system designed to monitor key parameters of medium- and high-voltage batteries in hybrid and electric vehicles. The system captures and analyzes voltage, current, temperature, and accelerator angle through sensors embedded in the electric propulsion system to calculate the SOC. It performs continuous monitoring to ensure adaptability under challenging driving conditions, such as the mountainous and rainy terrain of Ecuador’s Sierra region.

The proposed system integrates high-precision sensors with intelligent prediction algorithms that collect and process data to deliver accurate SOC estimations. By doing so, it reduces the errors typically associated with generalized methods for estimating SOC and driving range in electric vehicles. The model explicitly considers environmental and operational factors, such as temperature, discharge rates, and driver acceleration behavior to support more efficient energy management strategies.

The monitoring system features a modular architecture composed of high-precision sensors, a microcontroller, and actuators for real-time data visualization and storage. In addition, the software layer employs machine learning techniques and predictive models to estimate the SOC of medium- and high-voltage batteries based on the data acquired from the system hardware.

### 3.1. Hardware

The hardware component consists of a direct current (DC) circuit in which the power battery is connected to the load devices through a protection circuit that ensures stable and safe operation. Sensor data are transmitted wirelessly to the control board via dedicated communication links.

**Figure 5:**
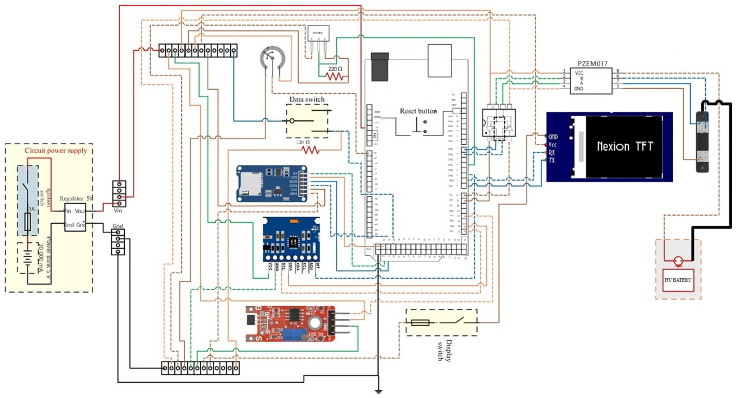
Electrical connections of the medium- and high-voltage battery monitoring system.

Figure 5 illustrates the electronic schematic of the motherboard where the monitoring sensors are installed. This diagram shows the interconnections between the sensors, microcontroller, and actuators. The motherboard collects data from the sensors, records the information, and uses it to calculate the battery’s state of charge.

Among the integrated sensors is the voltage and current sensor (5), specifically the PZEM-017 DC sensor, which is designed for simultaneous voltage and current measurement in electric propulsion systems. Its operation is based on detecting voltage differentials and monitoring current flow through the circuit, sending the data to an RS485 communication module for digital processing. This sensor is manufactured by Jeanoko Co., Ltd., located in Shenzhen, China, a company specializing in energy instrumentation and monitoring.

The PZEM-017 is essential for monitoring medium- and high-voltage batteries, as it enables real-time analysis of energy consumption and supports accurate SOC estimation. Its measurement range spans from 0.05 to 300 V with a resolution of 0.01 V, and from 0.02 to 300 A for current, with a resolution of 0.01 A.

The DS1820 digital temperature sensor (3), manufactured by Maxim Integrated headquartered in San Jose, CA, USA (now part of Analog Devices, Wilmington, MA, USA), is widely recognized for its reliability and ease of integration in electronic thermal monitoring systems. It operates on a single-wire bus protocol, which simplifies wiring and enhances system scalability—particularly valuable in complex configurations such as battery packs. With an accuracy of ±0.5 °C and typical operating voltages of 3.3 V or 5 V, the DS1820 provides stable and accurate thermal measurements, making it well-suited for temperature monitoring in electric vehicle power systems.

The 100K potentiometer (2), commonly used for throttle position measurement, is a standard component in analog control systems and is supplied by established manufacturers such as ALPS Alpine Co., Ltd., based in Tokyo, Japan. It operates within a voltage range of 3.3 V to 5 V. The device functions based on resistance variation as the throttle is actuated, generating an analog voltage proportional to the rotation angle. This signal is then digitized by an analog-to-digital converter (ADC), allowing the system to accurately interpret the driver’s power demand.

The MPU6050 accelerometer (12) is a six-degree-of-freedom inertial measurement unit (IMU) developed by InvenSense Inc., headquartered in San Jose, CA, USA (currently part of TDK Corporation, Tokyo, Japan). It combines a three-axis accelerometer with a three-axis gyroscope, enabling simultaneous measurement of linear acceleration and angular velocity (tilt or yaw). The MPU6050 operates with a supply voltage between 3 V and 5 V and communicates via an I^2^C interface, providing updated measurements at approximately 1000 ms intervals. Its high sensitivity and responsiveness to dynamic changes make it an excellent choice for applications requiring real-time vehicle stability monitoring under various operating conditions.

The microcontroller (4) operates at a clock frequency of 16 MHz and processes both analog inputs (voltage, current, and temperature) and digital inputs (acceleration and throttle angle). Temperature and throttle position sensors are connected via a 1-wire bus, facilitating simplified integration. Voltage and current measurements are routed through a differential-to-binary interface and transmitted over a 1-wire bus to the microcontroller’s digital input. A six-degrees-of-freedom (6-DoF) inertial measurement unit—combining a three-axis accelerometer and a three-axis gyroscope—monitors dynamic vehicle behavior and is connected through a three-wire bus. Vehicle speed is measured using a Hall effect sensor, and a microSD storage module is used to log data locally. 

A 5 V regulated power supply is provided by an LM7805 voltage regulator, sourced from Texas Instruments Inc., headquartered in Dallas, TX, USA, protected by a resettable fuse. RC filters with a cutoff frequency of 10 Hz are applied to analog inputs to suppress noise. Galvanic isolation between high-voltage sensors and the battery is achieved using optocouplers, ensuring system safety.

Sensor calibration is performed to ensure reliable and accurate data acquisition. Voltage sensors are calibrated using an adjustable DC power supply and a Trisco DA830 digital multimeter, manufactured by Trisco Technologies Corp., based in Taichung City, Taiwan, compensating for deviations across the operating range. Current sensors are calibrated similarly, using a precision current source and a reference ammeter to verify measurements throughout their full scale. Temperature sensors are calibrated using a Goyojo GW192 thermal imaging camera, manufactured by Goyojo Technology Co., Ltd., located in Shenzhen, China, and a reference thermometer, validating sensor outputs at multiple controlled temperature points. Throttle position sensors are calibrated using a stable voltage source and a digital multimeter to ensure accurate angular voltage correlation.

Finally, a 5-inch Nextion display is employed as a real-time visualization panel to present the SOC results. Through serial communication, the outputs from both predictive algorithms are integrated into the display to provide adaptive estimations under dynamic driving conditions. A computer program is embedded within the virtual interface to display key parameters related to the state of medium- and high-voltage batteries powering the electric traction system. The monitoring system was deployed on a test vehicle—a Super Soco electric motorcycle—for on-road testing and training data acquisition. SOC values from the medium-voltage battery were collected using standardized driving cycles, as detailed in Section 2.

### 3.2. Sensor Calibration and Reliability Assurance

To ensure accurate data acquisition, all embedded sensors were calibrated using laboratory-grade reference instruments prior to data collection.

A Fluke 87V True RMS multimeter was used as the reference for voltage and current measurements. It offers a DC voltage accuracy of ±0.05%+1 digit and a DC current accuracy of ±0.2%. Temperature calibration was conducted using a FLIR E5-XT thermal imaging camera, which provides a thermal sensitivity of <0.1 °C at 30 °C and an absolute accuracy of ±2 °C or ±2% of reading, whichever is greater.

Calibration involved applying different load profiles (no-load, partial load, and full load) and recording sensor readings in parallel with the reference devices. For temperature, thermal gradients were induced using forced air and heat sources to cover the expected operating range. To mitigate long-term sensor drift, a three-point recalibration protocol was applied every 30 days. Additionally, offset correction routines were implemented in firmware using idle-condition data to continuously recalibrate zero-current and zero-voltage baselines.

An experimental platform was implemented on a Super Soco electric motorcycle to perform real-time data acquisition and evaluate the accuracy of the proposed SOC estimation models. The vehicle was modified to integrate a custom monitoring system composed of various sensors and a microcontroller-based control unit.

**Figure 6:**
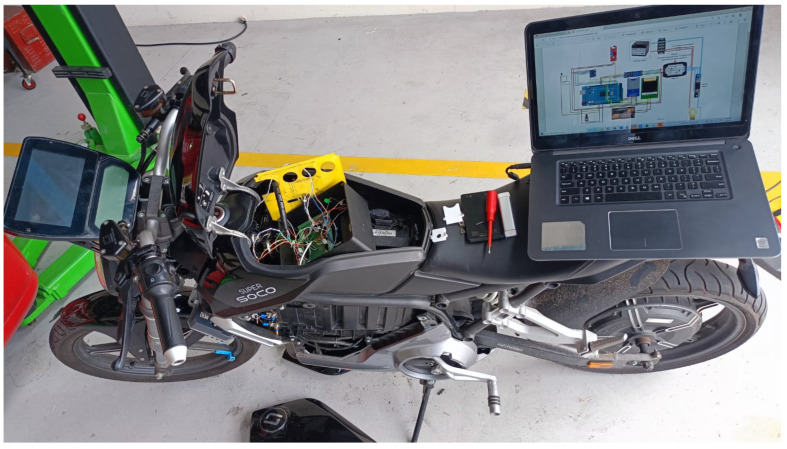
Experimental platform on electric motorcycle.

Figure 6 shows the modified motorcycle with the seat lifted, exposing the main components of the system. The control board (microcontroller) is installed beneath the seat, connected to the following sensors: a module for simultaneous voltage and current measurements, a digital temperature sensor, a throttle potentiometer, an inertial measurement unit, and a Hall-effect sensor for wheel speed measurement. All signals are routed to the control board through shielded cables to minimize electromagnetic interference during operation.

A laptop is placed on the rear seat to run the real-time monitoring software, which displays the system’s schematic and records the captured data. This setup allows for full supervision of sensor signals during on-road testing under standardized and custom driving cycles. The platform enables validation of both machine learning models—LSTM + GA and multiple linear regression—by capturing and comparing actual versus predicted SOC under varying conditions of load, terrain, and driving style.

### 3.3. Software

The software architecture integrates two predictive models: an LSTM neural network optimized using genetic algorithms, designed to capture temporal dependencies, and a MLR model to account for linear relationships among variables such as temperature, voltage, and current. Both algorithms update the estimated driving range every 0.385 s, combining high precision (at the sensor resolution level) with computational efficiency for dynamic operating environments.

Sensor signals are continuously fed into a regression system to capture operating variables in real time. The process begins with the acquisition of raw sensor data. If the reading is valid, the data are integrated into the software for interpretation and processing. These values are then displayed in real time on the visualization panel and stored on a microSD card via serial communication. In the event of an invalid or corrupted reading, the system discards the incoming signals and retains the last valid data stored in the memory unit. Finally, the validated signals are encoded in hexadecimal format for interpretation by the microcontroller using the implemented programming language.

**Figure 7:**
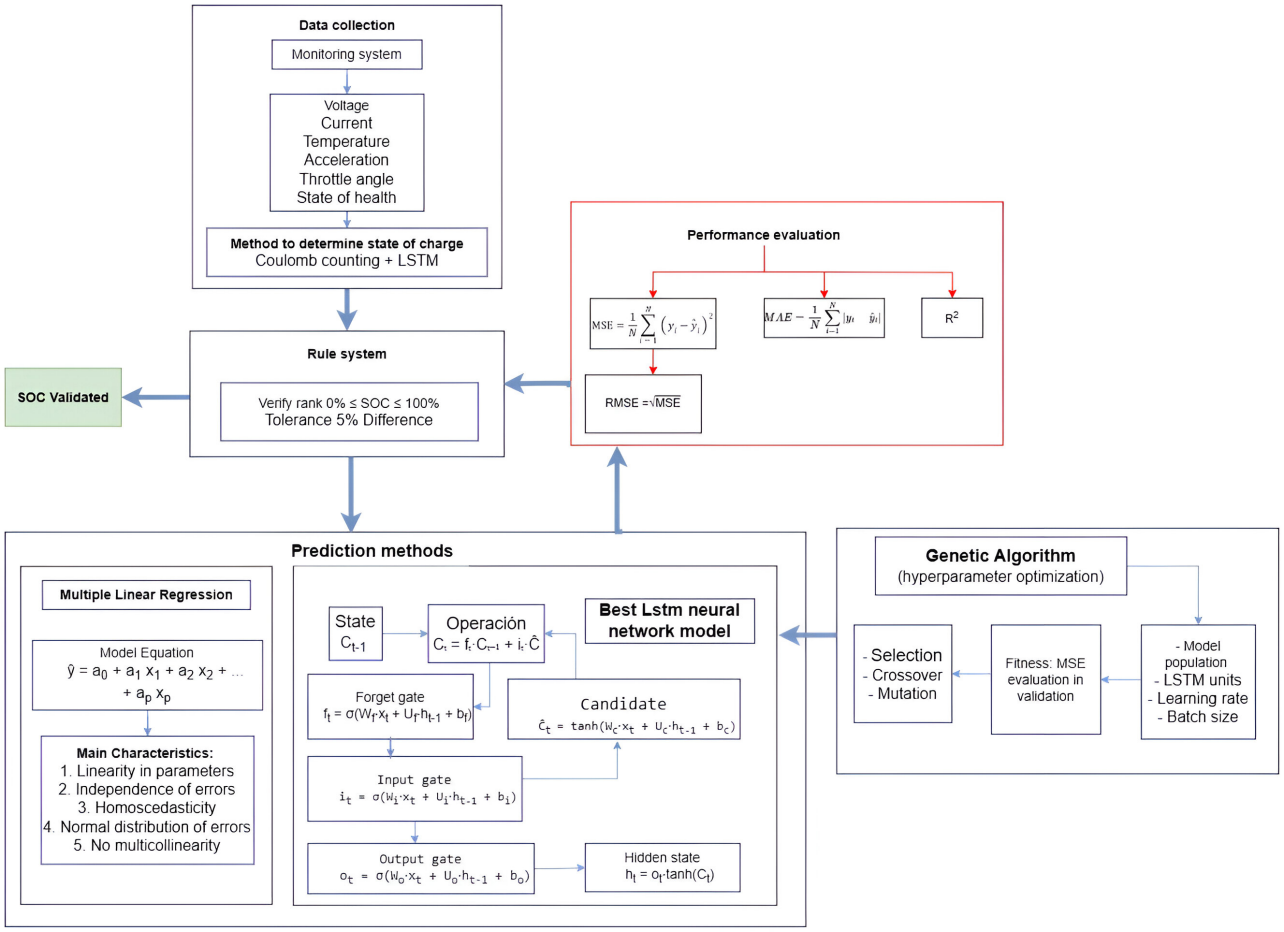
General flow diagram.

Figure 7 illustrates the software architecture of the integrated system designed to predict the state of charge in medium- and high-voltage batteries for electric vehicles. The system combines an LSTM neural network for predictive modeling and a MLR model to enhance accuracy based on the historical state of charge, denoted as SOC(t). Both models operate in parallel to provide robust and adaptive SOC estimation.

The MLR model includes operating parameters such as voltage, current, and temperature, producing an ${\mathrm{SOC}}_{\mathrm{pred}}$ (MLR). Meanwhile, the LSTM model structures its prediction through dynamic computations: the “state computer” observes the influence of noise and feeds the saturation of the cell $C_{i}$ into the hidden-layer output. The “output computer” then determines the hidden-layer output, yielding ${\mathrm{SOC}}_{\mathrm{pred}}$ (LSTM).

This technique chains recurrent regressors with input computers ($x_{i}$) and employs forget gate mechanisms to capture temporal dependencies. Genetic optimization is used to adjust hyperparameters (LSTM units, learning rate, and batch size) within a broad search space, using the mean squared error as the fitness function.

### 3.4. Long Short-Term Memory (LSTM) Neural Networks Optimized via Genetic Algorithms

As described in Section 2, this research employs long short-term memory neural networks optimized using genetic algorithms (GA). The LSTM network equations are defined in Equations (2)–(7), while the genetic algorithm-based optimization process is detailed in Equations (9)–(13). The LSTM network processes the time-series data corresponding to the SOC of medium- and high-voltage batteries using cells composed of three gates—forget, input, and output—and a cell state. Mathematically, an LSTM cell performs the following operations at each time step *t*.

This approach leverages the capacity of LSTM networks to model sequential data, while employing genetic algorithms to optimize critical hyperparameters such as the number of LSTM units, learning rate, and batch size. The model was trained using a dataset obtained from actual measurements of the WLTP urban driving cycle. Prior to training, the data were normalized to promote convergence and structured into sequences with five time steps to match the LSTM input format.

The LSTM network architecture incorporates advanced features, including optional bidirectional layers to capture temporal dependencies in both forward and backward directions, multiple LSTM layers with decreasing numbers of units to reduce computational redundancy, and an adjustable dropout mechanism to mitigate overfitting. The framework is flexible, allowing customization of time window lengths (ranging from 5 to 30 steps) and batch sizes (ranging from 8 to 128) to accommodate sequences of varying length and complexity.

Hyperparameter optimization is performed using genetic algorithms, with a search space encompassing seven parameters: number of LSTM layers (1–3), number of units in the first layer (16–128), dropout rate (0–0.5), use of bidirectionality (binary), learning rate (0.0001–0.01), time step window, and batch size. A population size of 30 individuals and 40 generations are employed to thoroughly explore the solution space. Custom genetic operators are implemented to ensure an effective balance between exploration and exploitation. Combined with temporal cross-validation and a fixed random seed for reproducibility, this strategy enhances both the robustness and generalization capability of the model.

Model validation was performed using a 70–30% training–testing data split. Performance was evaluated using standard metrics, including MSE, RMSE, MAE, and the coefficient of determination ($R^{2}$). To prevent overfitting, an early stopping mechanism based on validation loss was employed during training. The synergy between the LSTM network responsible for capturing temporal patterns and genetic algorithm based optimization used for fine tuning hyperparameters enabled accurate SOC prediction in electric vehicles. This hybrid approach demonstrated strong performance under both standardized WLTP driving cycles and custom-defined test routes.

### 3.5. Multiple Linear Regression Model Formulation

This study also implements a multiple linear regression model to estimate the state of charge of a battery based on operational data. The model’s performance is assessed using standard evaluation metrics, including mean squared error, mean absolute error, and root mean squared error. In addition, scatter plots and correlation matrices are presented to support interpretation and assess the model’s predictive effectiveness. The dataset obtained from road tests serves as the training input for the application of Equations (14)–(21), which relate SOC to the selected independent variables.

The MLR model estimates SOC using temporal data on current, voltage, and time. The dataset is partitioned into training (70%) and testing (30%) subsets, with a fixed random state to ensure reproducibility. The model is trained and evaluated using MSE, RMSE, MAE, and the coefficient of determination ($R^{2}$), highlighting its explanatory capability. Additionally, the model includes visualization tools for comparing actual vs. predicted values, analyzing residuals, and displaying the correlation structure among input features. Robustness is further assessed through five-fold cross-validation, which computes the average MSE across folds.

The hyperparameters used in this methodology include a test size of 0.3, indicating that 30% of the data are allocated for testing and 70% for training. A random state of 42 is applied to ensure consistent data splitting. The number of cross-validation folds is set to 5, and the negative mean squared error is used as the scoring metric during cross-validation.

### 3.6. Rule System

As shown in Figure 7, the flowchart of the SOC monitoring and prediction system for electric vehicle batteries includes a rule-based validation module that plays a critical role in verifying the plausibility of model outputs. This system ensures that both the actual and predicted SOC values fall within the acceptable range of 0% to 100%, with an allowable deviation of up to 5% between the two. The rule engine serves as a safety filter, validating the results generated by both the multiple linear regression and LSTM models to ensure consistency with the physical constraints of the battery.

If any predicted SOC value lies outside the valid range, such as negative values, values exceeding 100%, or significant disagreement between the models, the system automatically triggers an alert. This functionality enables early intervention to prevent battery degradation, incorrect energy management decisions, or errors in vehicle range estimation.

The rule-based system also defines several critical thresholds to enhance operational safety and diagnostic reliability:

- A maximum allowable deviation of 5% between actual and predicted SOC values,
- A defined safe operating SOC range between 20% and 95%,
- A threshold for anomalous rates of change exceeding 2% per minute.

Alarms are triggered when these limits are exceeded, when performance metrics indicate high error (e.g., MSE > 0.2 or MAE > 3%), or when model overfitting is detected (defined as a difference greater than 0.25 $R^{2}$ between training and test sets).

Automated corrective actions include: (i) model recalibration after three critical alarms within one hour, (ii) immediate operator notification if the SoC exits the safe range, and (iii) detailed logging of all anomalies for post-analysis and model refinement.

The ±5% criterion was selected based on the following considerations:

1. In commercial battery management systems (BMS), this margin is widely accepted as a practical trade-off between accuracy and operational safety. It accounts for typical sensor noise, cell-to-cell variability, and system tolerance, while minimizing the occurrence of false alarms.
2. Benchmark studies in the SOC estimation literature report acceptable tolerances ranging from 3% to 7% for electric vehicle (EV) applications. A 5% threshold ensures compliance with these standards, preserving battery longevity and user safety without imposing unrealistic precision demands.
3. The threshold aligns with the effective resolution of the measurement system used in this study (based on sensors with an accuracy of ±0.5–1%) while also incorporating a margin for modeling uncertainties. Reducing the threshold further would offer limited practical benefit and could increase the likelihood of triggering unnecessary alerts.

## 4. Results

Accurate estimation of the state of charge in medium- and high-voltage battery systems remains a critical challenge for ensuring the efficient and safe operation of electric vehicles. In this study, LSTM neural networks optimized using genetic algorithms are employed to predict SOC based on key variables related to battery performance. This hybrid approach leverages the sequential modeling capabilities of LSTM networks, while the genetic algorithm is used to fine-tune essential hyperparameters, including the number of LSTM units, learning rate, and batch size.

The training dataset consists of samples collected from standardized driving cycles, such as WLTP and NEDC, as well as a custom driving route designed to reflect real-world conditions. The historical SOC of the test vehicle’s battery was obtained through extensive road testing and is used as the ground truth target for the supervised learning models developed in this work.

### 4.1. Experimental Setup

Two types of lithium-ion battery packs were used in the experimental validation:

- Medium-voltage battery: A 60 V, 40 Ah NCM lithium-ion pack extracted from a Super Soco electric motorcycle (manufactured by Vmoto Soco Group, Perth, Australia). It consists of 16 cells in series and was selected for its accessibility, manageable voltage level, and suitability for real-time onboard data acquisition.
- High-voltage battery: A 240 V, 6.5 Ah pack (64 cells) repurposed from a second-generation Kia Niro EV (manufactured by Kia Corporation, Seoul, Republic of Korea). This battery was used for testing the model’s generalization under WLTP and NEDC cycles in a controlled laboratory environment.

The data acquisition system was built around an Arduino Mega 2560 microcontroller equipped with INA219 voltage/current sensors, a GPS module, a throttle position sensor (0–5 V analog input), a Bosch MPU6050 accelerometer and an I2C temperature sensor. The SOC reported by the original BMS was recorded for reference.

Sampling Configuration: All signals were sampled at a frequency of 2.6 Hz (approximately every 0.385 s) and stored on a microSD card in CSV format for offline processing. The acquisition included the following signals:

- Battery voltage (V),
- Battery current (A),
- Battery surface temperature (°C),
- GPS-based vehicle speed (km/h),
- Throttle angle (0–100%),
- Longitudinal acceleration (m/s^2^).

Data were collected during predefined driving cycles (NEDC and WLTP) and a custom inter-campus route. Before each test, sensors were calibrated using laboratory references (see Section 3.2). This detailed configuration ensures the reproducibility of the measurements and the validity of the SOC estimation results.

### 4.2. Experimental Protocols for Dynamic and Static Testing

The SOC estimation models were validated using two complementary experimental approaches: dynamic tests with an electric motorcycle and static discharge tests with high-voltage batteries, each following NEDC and WLTP driving cycle standards.

#### 4.2.1. Dynamic Tests: Super Soco Electric Motorcycle

Dynamic tests were carried out using a Super Soco electric motorcycle equipped with GPS, current and voltage sensors, temperature probes, and throttle position sensors. These tests were conducted at the Universidad de las Fuerzas Armadas ESPE—Campus Belisario Quevedo.

- Urban NEDC cycle: Distance of 11 km, speed range of 0–34 km/h, estimated test duration of 20 min.
- Extra-urban NEDC cycle: Distance of 11 km, speed range of 0–120 km/h, estimated duration of 10–20 min.
- WLTP urban cycle: Distance of 23.25 km, maximum speed of 46.5 km/h, average duration 30 min.
- WLTP extra-urban cycle: Distance of 23.26 km, speed range 0–120 km/h, average duration 20–30 min.

Each test was performed twice, under dry conditions, average ambient temperatures between 21 °C and 26 °C, and low wind. The terrain was asphalted and level. Data was acquired at 2.6 Hz.

#### 4.2.2. Static Battery Testing: High-Voltage Pack

A 240 V, 7 Ah high-voltage lithium-ion pack was tested in a laboratory environment using a programmable DC electronic load. The battery was subjected to current profiles that emulate WLTP and NEDC cycles. No driver was required; tests were executed on a stand in zero-load mechanical condition (no real torque).

- Five test repetitions were conducted for each profile.
- The system monitored voltage, current, temperature, SOC, and acceleration.
- Motor rotation was registered from 0 to 600 wheel revolutions, with no physical traction load.

These controlled tests allowed repeatable capture of battery response under pseudo-realistic profiles and ensured safe characterization of the SOC estimation model.

### 4.3. SOC Prediction for Each Driving Cycle

This study presents a comparative analysis of state-of-charge estimation results for an electric motorcycle subjected to five distinct driving profiles. These include: (i) the NEDC urban cycle, (ii) the NEDC extra-urban cycle, (iii) the WLTP urban cycle, (iv) the WLTP extra-urban cycle, and (v) a custom driving cycle simulating inter-campus travel between the two locations of the Universidad de las Fuerzas Armadas ESPE in Latacunga.

For each scenario, temporal curves comparing predicted vs. actual SOC values are provided, along with quantitative performance metrics (MSE, RMSE, MAE and $R^{2}$) generated by the LSTM model optimized through a genetic algorithm.

This analysis underscores how variations in speed, acceleration, and battery operating conditions across different driving cycles impact the accuracy of state-of-charge estimation. It enables the identification of conditions under which the predictive algorithm exhibits enhanced robustness, as well as scenarios where refinements to the battery management system (BMS) may be required to improve SOC prediction performance.

**Figure 8:**
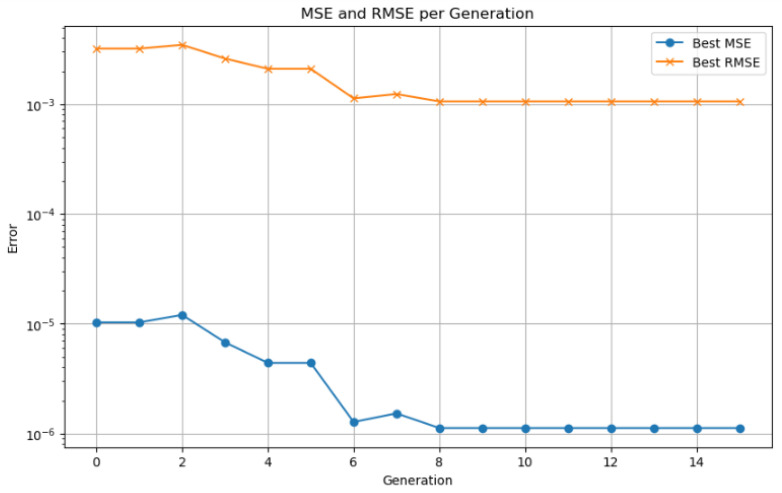
Evolution of MSE and RMSE during the urban NEDC cycle.

The LSTM hybrid model, optimized using a genetic algorithm, demonstrated rapid convergence. The scaled MSE was reduced from $1\times 10^{-5}$ in the initial generation to $1.12\times 10^{-6}$ by the sixth generation. This optimization resulted in an almost perfect alignment between the predicted and actual SOC curves during the NEDC urban cycle, as illustrated in Figure 8. In this chart, each point along the X-axis represents a generation of the genetic algorithm. A generation includes the selection, crossover, and mutation steps used to produce a new population of hyperparameter vectors, followed by the evaluation of an LSTM model trained with each of them.

In absolute terms, the model achieved an MSE of 0.0054, an RMSE of 0.0736, a MAE of 0.0685, and a coefficient of determination of $R^{2}=0.9987$, reflecting its high precision and reliability in SOC estimation tasks under real-world urban driving conditions.

**Figure 9:**
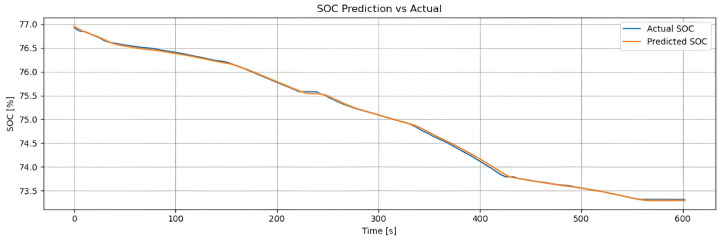
Comparison between predicted SOC and actual SOC during the urban NEDC cycle.

Figure 9 illustrates that the SOC curve predicted by the model (orange line) closely tracks the actual SOC evolution (blue line) throughout the NEDC urban cycle. The minimal discrepancies demonstrate the model’s exceptional tracking accuracy. This near-perfect alignment confirms that the hyperparameters optimized via the genetic algorithm enabled the LSTM model to accurately learn and replicate the battery’s discharge behavior.

**Figure 10:**
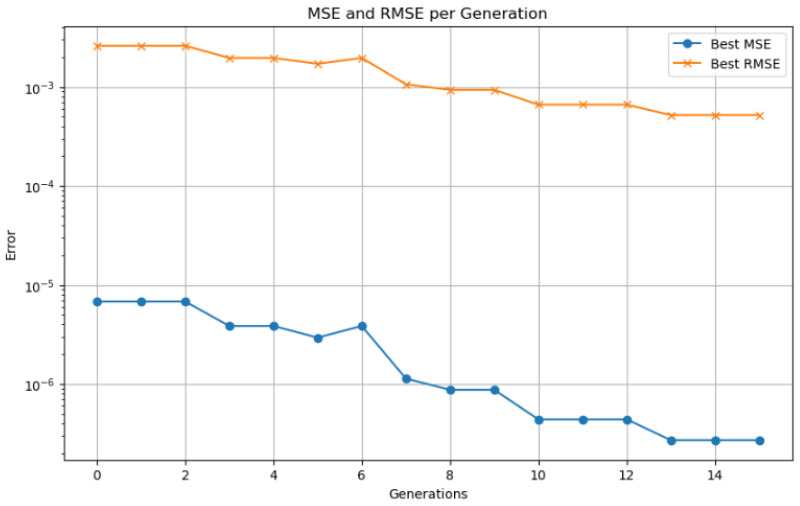
Evolution of MSE and RMSE during the extra-urban NEDC cycle.

Figure 10 presents the analysis of the NEDC extra-urban cycle. During the training phase, a rapid reduction in the training MSE is observed within the first five epochs, reaching values close to zero. The validation loss, after a slight peak around the third epoch, also converges to a minimum level with no signs of overfitting. This behavior, together with the final performance metrics (original MSE = 0.00076, RMSE = 0.0275, MAE = 0.0232, and $R^{2}=0.9912$), confirms the effectiveness and generalization capability of the model.

**Figure 11:**
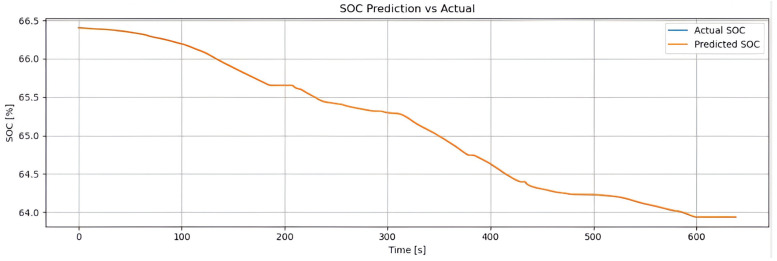
Comparison between predicted SOC and actual SOC during the extra urban NEDC cycle.

Figure 11 compares the actual and predicted SOC values obtained by the LSTM + GA model during the NEDC extra-urban cycle. The near-perfect overlap of both curves, with maximum deviations below 0.1%, demonstrates that the model accurately reproduces the battery discharge dynamics under extra-urban driving conditions. These results, supported by a coefficient of determination of $R^{2}=0.9912$ and RMSE = 0.0275, confirm the accuracy of the proposed approach.

**Figure 12:**
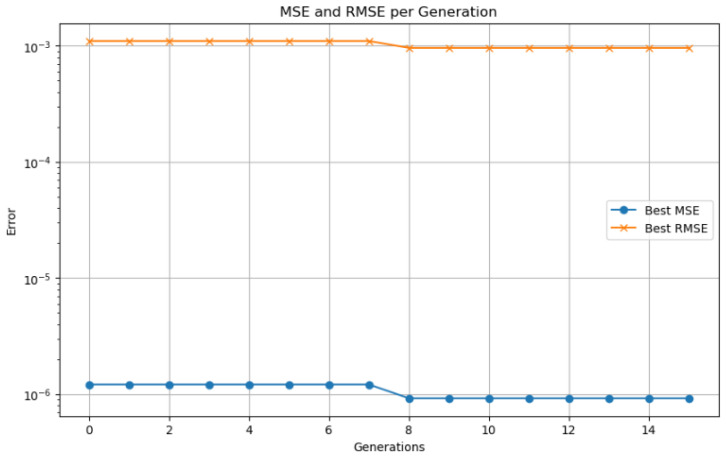
Evolution of MSE and RMSE during the urban WLTP cycle.

Figure 12 presents the analysis of the WLTP cycle conducted in a low-speed urban circuit. The LSTM-GA model achieved an RMSE of 0.3942 and an MSE of 0.1554. The predicted curve (orange line) closely follows the actual SOC curve (blue line).

**Figure 13:**
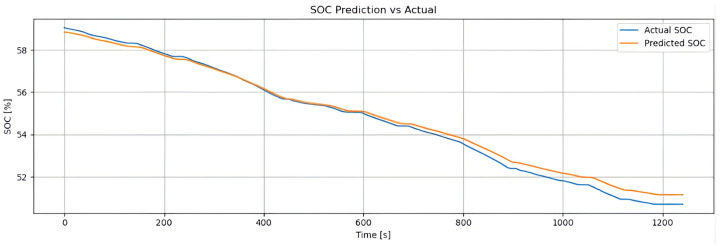
Comparison between predicted SOC and actual SOC during the urban WLTP cycle.

Figure 13 compares the actual and predicted SOC values obtained by the LSTM model optimized with a genetic algorithm throughout the WLTP urban cycle. The curves exhibit a very close match during each acceleration and braking phase, with maximum deviations below 0.5% of the SOC. This demonstrates the model’s ability to accurately reproduce the battery discharge dynamics under urban traffic conditions. These results, supported by a coefficient of determination of $R^{2}=0.9925$ and an original RMSE of 0.3942% SOC, confirm the accuracy of the proposed approach.

**Figure 14:**
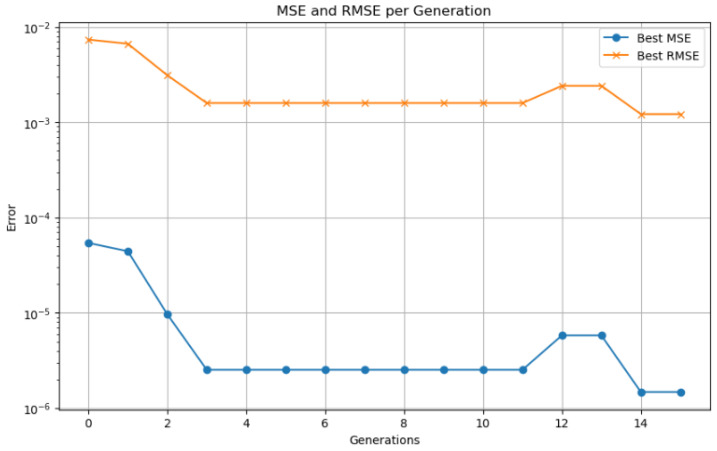
Evolution of MSE and RMSE during the extra urban WLTP cycle.

Figure 14 shows the evolution of prediction errors, highlighting how the genetic algorithm rapidly reduces the MSE from $4\times 10^{-5}$ in the initial generation to a stable value of $1\times 10^{-6}$ from the fourth generation onward. Meanwhile, the RMSE decreases from $8\times 10^{-3}$ to $1.3\times 10^{-3}$, with only a slight increase in generation 12. This behavior demonstrates the convergence and stability of the LSTM model optimized with a genetic algorithm. The final results (original MSE = 0.0468, RMSE = 0.2163% SOC, MAE = 0.1637%, and $R^{2}=0.9908$) confirm that the model delivers accurate SOC estimation for the WLTP extra-urban cycle, with high precision and a mean absolute error below 0.17%.

**Figure 15:**
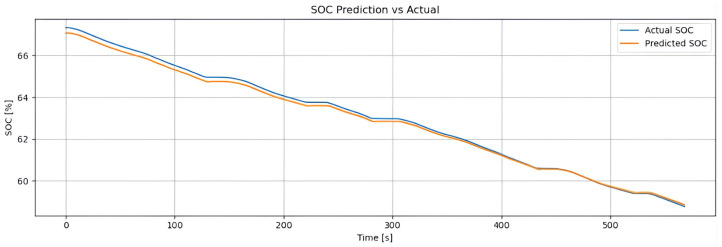
Comparison between predicted SOC and actual SOC during the extra urban WLTP cycle.

Figure 15 presents the comparison between actual and predicted SOC throughout the WLTP extra-urban cycle. The results show a very close overlap between the two curves, covering approximately 67% to 59% of the SOC range. The maximum discrepancies, below 0.3%, occur mainly during transitions in driving conditions, indicating that the proposed approach effectively captures the uniform battery discharge dynamics characteristic of open-road driving.

**Figure 16:**
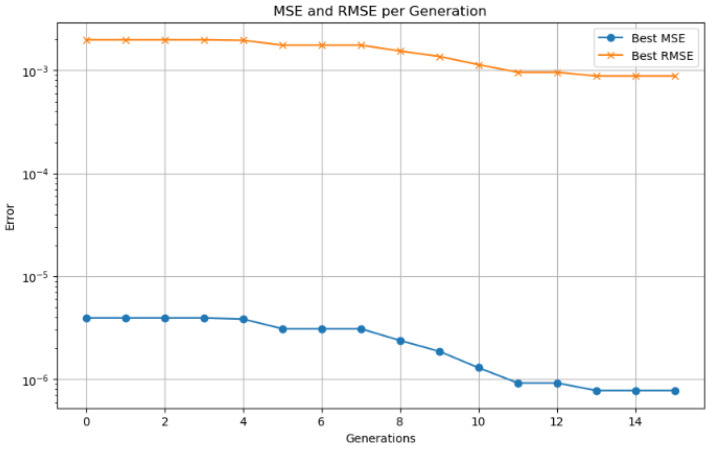
Evolution of MSE and RMSE during the inter-campus cycle.

Figure 16 shows the evolution of prediction errors across generations of the genetic algorithm applied to the LSTM model for the custom inter-campus driving cycle at ESPE Latacunga. The final performance metrics in the original scale are as follows: MSE = 0.0047, RMSE = 0.0682% SOC, MAE = 0.0614%, and $R^{2}=0.9986$. These values confirm that the optimized model achieves high precision in estimating the battery’s state of charge under real-world inter-campus driving conditions.

**Figure 17:**
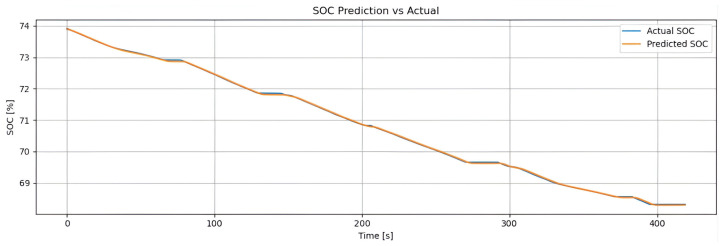
Comparison between predicted SOC and actual SOC during the inter-campus cycle.

Figure 17 compares the actual and predicted SOC obtained by the LSTM model optimized with a genetic algorithm during the inter-campus driving cycle. The maximum deviations, all below 0.1%, demonstrate that the model accurately captures the battery discharge dynamics under real-world driving conditions. The final quantitative metrics confirm the high precision of the LSTM + GA approach.

Table 6, summarizes the main error metrics (MSE, RMSE, MAE) and the coefficient of determination ($R^{2}$) for the state-of-charge estimator based on an LSTM model optimized using genetic algorithms, evaluated across five representative driving cycles (urban, extra-urban, mixed, and custom). The last row shows the average values, providing a clear comparison of the model’s accuracy and robustness under different driving conditions.

**Figure 18:**
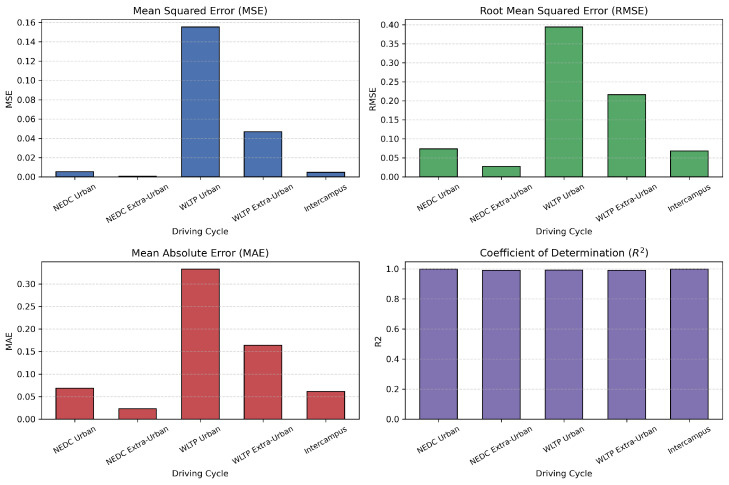
Comparative view of the metrics obtained.

Figure 18, presents a comparative view of the four key metrics obtained using the LSTM model optimized by a genetic algorithm across the five driving profiles studied: NEDC urban, NEDC extra-urban, WLTP urban, WLTP extra-urban, and inter-campus. This combined visualization allows for a quick assessment of how the accuracy of the SOC estimator varies according to the dynamic complexity of each cycle. While urban routes (NEDC and WLTP) exhibit very low errors and $R^{2}$ values close to unity, the cycles with greater speed fluctuations (e.g., WLTP urban) show higher error peaks. Meanwhile, the inter-campus profile demonstrates excellent performance with minimal deviations, confirming the robustness and versatility of the proposed approach for real-world applications.

The color scheme in the bar charts is used to distinguish the different evaluation metrics: blue for MSE, green for RMSE, red for MAE, and purple for ($R^{2}$).

### 4.4. Performance Comparison: Baseline LSTM vs. GA-Optimized LSTM

To quantify the impact of genetic algorithm optimization on model performance, we compared two configurations:

- Baseline LSTM: Configured with conventional hyperparameters from literature: 2 layers, 100 hidden units per layer, learning rate = 0.001, batch size = 32, optimizer = Adam.
- GA-Optimized LSTM: Hyperparameters automatically selected via genetic algorithm with fitness function based on validation MAE.

Both models were trained and evaluated using the same dataset and preprocessing steps. Table 7 summarizes the comparative results:

As observed, the GA-optimized model achieves a 63.89% reduction in MAE and 66.7% in RMSE compared to the manually tuned baseline. Although training time increases due to the evolutionary search, the accuracy gains justify the additional computational cost in offline applications.

### 4.5. Multiple Linear Regression Results

The multiple linear regression model assumes a direct linear relationship between the independent variables and the state of charge, which may not fully capture system behavior under nonlinear conditions such as abrupt traffic variations, temperature fluctuations, or complex terrain profiles. Combined effects of environmental and operational factors (temperature, current flow, and road gradient) may not be adequately represented, leading to minor inaccuracies in SOC estimation. Although the model was trained using data collected under real-world driving conditions, its predictive accuracy may degrade when applied to markedly different or extreme scenarios.

**Figure 19:**
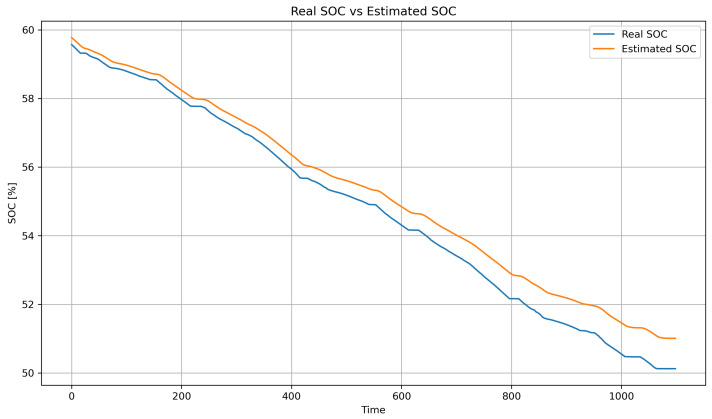
Real and predicted state-of-charge values by multiple linear regression.

Figure 19, which compares actual and predicted SOC values using the MLR model, shows good agreement in overall trend, particularly in the high SOC range (55–60%), where predictions closely match actual values. However, notable deviations are observed in the mid-to-low SOC ranges (50–55%), especially during sharp declines in the actual SOC curve. These discrepancies suggest limitations of the linear model in capturing nonlinear dynamics or rapid discharge events. The presence of fluctuations in the actual SOC values may indicate sensor noise or external influences not accounted for in the model, while the slight prediction lag reflects unmodeled temporal dependencies.

**Figure 20:**
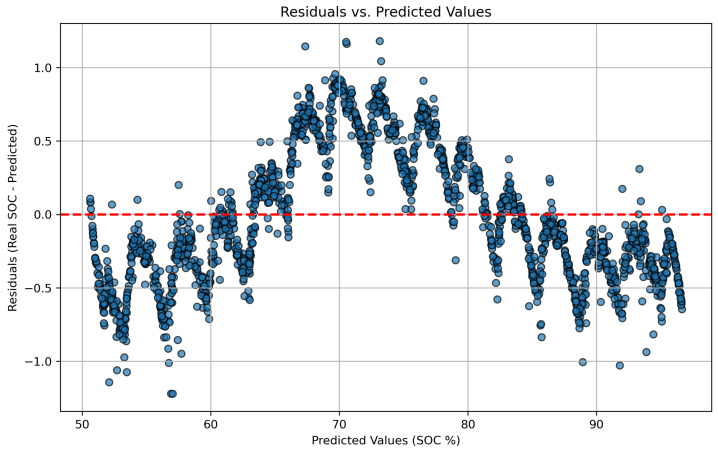
Waste distribution.

Figure 20, presents the distribution of residuals (i.e., the differences between actual and predicted SOC values) as a function of the predicted SOC, with the x-axis spanning from 70% to 95%. In the high SOC range, residuals are relatively small and randomly distributed around the zero line, indicating a good fit and minimal bias in this region.

However, in the intermediate range, greater dispersion and emerging systematic patterns are observed. Several negative residuals suggest that the model consistently overestimates the actual SOC in this segment. Furthermore, the presence of a few outliers in the low SOC range indicates substantial prediction errors during deep discharge conditions.

While the overall residual variance shows no strong trend, suggesting partial homoscedasticity, the systematic underestimations in the mid-range highlight the limitations of the linear model in capturing nonlinear battery behavior under varying operational conditions.

The following performance metrics were computed to evaluate the predictive capability of the multiple linear regression model:

- MSE: 0.9457, which quantifies the average of the squared differences between actual and predicted values. Lower values indicate a better model fit.
- RMSE: 0.9725, expressed in the same units as the target variable (SOC), offering an interpretable scale for assessing prediction error.
- MAE: 0.8851, which represents the average of the absolute differences between predicted and actual values. Like RMSE, a low MAE reflects high prediction accuracy.

These metrics confirm that the MLR model produces low prediction errors, demonstrating its capacity to capture the underlying relationships between the independent variables and the SOC. The close alignment between predicted and actual values reinforces the model’s strong explanatory power. The overlap observed at most time points indicates a high level of agreement and a low margin of deviation.

Cross-validation yielded an MSE of 0.8941, calculated as the average of the mean squared errors across multiple folds of the training set. This value is consistent with the MSE obtained on the test set (0.9457), confirming that the model generalizes well and does not suffer from overfitting or underfitting.

Furthermore, the coefficient of determination, $R^{2}=0.9534$, indicates that approximately 95.34% of the variability in SOC is explained by the model. This high $R^{2}$ value demonstrates a strong goodness-of-fit and confirms that the MLR model reliably captures the key dependencies in the dataset.

## 5. Discussion

This study analyzes and compares the accuracy of two approaches for estimating the state of charge in electric vehicle batteries: a hybrid LSTM model optimized using genetic algorithms, and multiple linear regression. The objective is to assess the robustness and applicability of each method using standard metrics (MSE, RMSE, MAE, and $R^{2}$) across five representative test scenarios.

The results show that the LSTM + GA model achieved an average MSE of 0.0426, an average RMSE of 0.1559, and an average $R^{2}$ of 0.9944, demonstrating its ability to capture both the general trend and nonlinear fluctuations of SOC across diverse driving cycles (urban, extra-urban, mixed, and custom). The average MAE of 0.1299% confirms a low mean deviation, making it suitable for battery management applications where accuracy is critical. In contrast, the MLR model exhibited a significantly higher average error (MSE around 0.9457 and RMSE of 0.9725% in global tests), although it retains the advantages of simplicity and interpretability.

Compared to a standalone LSTM model, the hybrid LSTM + GA approach offers clear advantages in terms of automated hyperparameter optimization. By evolving network architecture and learning rate parameters through genetic operations, it typically achieves higher prediction accuracy and faster convergence during training, without the need for extensive manual tuning. However, these benefits come at the cost of increased computational complexity and longer training times, as each GA generation requires multiple fitness evaluations of the LSTM network.

In the context of embedded battery management systems, where computational resources are limited and real-time constraints are stringent, such overhead may necessitate offline optimization phases or the use of dedicated hardware accelerators to meet latency and power constraints. In contrast, a standalone LSTM implementation significantly lower computational cost and is easier to deploy, though it may result in suboptimal accuracy and require expert intervention. Therefore, choosing between a standalone LSTM and an LSTM + GA hybrid model involves a trade-off between prediction performance and the cost, latency, and maintainability requirements of the target BMS application.

Overall, these averages indicate that while both methods are viable, the LSTM + GA approach offers substantial improvements in accuracy under dynamic and nonlinear conditions. In high energy demand scenarios, the GA-tuned LSTM network achieves near-perfect tracking of the actual SOC, while the MLR model tends to smooth out peaks and valleys. For embedded BMS systems where computational resources are sufficient, the LSTM + GA approach is recommended to ensure more reliable SOC estimations. In contrast, for hardware-constrained environments or applications where model transparency is prioritized, MLR remains an acceptable alternative, particularly under more linear discharge cycles.

Multiple linear regression remains a valuable and robust tool despite its lower accuracy, especially in scenarios where simplicity, interpretability, and lower computational resource requirements are valued. The selection of the method depends on the system’s priorities. For critical and complex applications, such as real-time monitoring and advanced battery management, the recommended choice is LSTM + GA. For preliminary tasks or scenarios where interpretability and computational efficiency are essential, MLR may be sufficient. This research validates the potential of optimized neural networks as key tools in advanced predictive systems, opening new possibilities to improve energy management in electric vehicles and other industrial applications.

The proposed GA-LSTM-based SOC estimator introduces several innovations that go beyond existing approaches. First, by combining genetic algorithms with LSTM neural networks, the method automatically optimizes hyperparameters such as layer size, learning rate, and dropout, improving model generalization and convergence stability. This represents a qualitative improvement over conventional LSTM implementations, which often require manual tuning and are prone to overfitting.

Second, compared to traditional regression techniques like MLR, the proposed method exhibits significantly higher predictive accuracy, with RMSE and MAE values reduced by more than 66.7% and 63.89%, respectively, under real-world EV data conditions. While MLR and standard machine learning models offer simplicity and fast computation, they struggle to capture the nonlinear temporal dependencies inherent in dynamic battery behavior.

Third, unlike Kalman-filter-based estimators or model-dependent techniques, the GA-LSTM approach is entirely data-driven, and therefore it does not rely on precise parameter identification or prior modeling assumptions—making it more adaptable to different battery chemistries, voltage ranges, and load profiles.

Finally, the model demonstrates robustness to input variability, including variations in temperature, load current, and initial SOC. These features address major challenges of SOC estimation in medium- and high-voltage battery systems, which are frequently exposed to rapid dynamic conditions in electric vehicles and hybrid systems.

Under dynamic operating conditions, such as those encountered during real-world driving or high-frequency load changes, several physical and electrical phenomena can affect the accuracy of SOC estimation. Rapid current fluctuations can lead to voltage drops due to internal resistance, introducing transient behaviors that mask the actual battery state. Simultaneously, battery hysteresis, polarization effects, and temperature variations further complicate the voltage–SOC relationship, making linear or steady-state models insufficient. These conditions can also introduce significant noise and delay in voltage and current measurements, reducing the effectiveness of traditional estimation methods that rely on static assumptions or simplified equivalent circuit models. The GA-LSTM approach used in this study addresses these challenges by learning temporal patterns from real operational data, allowing the model to adapt to dynamic behavior and preserve accuracy despite the presence of nonlinearity, measurement noise, and time-varying system dynamics.

Although the application of machine learning (ML) to the classification and analysis of motion capture data is a relatively recent research field, several studies align with the present work in areas such as state-of-charge estimation, emerging sensor technologies, and physics-guided methodologies for lithium battery monitoring.

In their study, Obuli et al. [8] evaluate a range of machine learning algorithms, including support vector machines, neural networks, ensemble methods, and Gaussian process regression, to estimate the SOC of electric vehicle batteries using real-time driving data. Among the methods assessed, Gaussian process regression demonstrated superior accuracy and lower error rates in experiments involving real-world data, accounting for variables such as driver behavior, ambient temperature, and driving cycles. The case studies validated the algorithm’s capacity to replicate actual charge/discharge characteristics, providing a robust and reliable framework for real-time battery monitoring and optimization within battery management systems.

A study conducted by Pamula et al. [38] addressed energy consumption prediction in electric vehicles using deep learning models that utilize basic operational data (routes, schedules, altitude). After evaluating DLNA, MLP, LSTM, and linear regression, the DLNA network demonstrated the highest accuracy (MAPE: 6.2%), even outperforming complex analytical methods. The study revealed that adding parameters improves accuracy by 1% and that linear regression (MAPE: 7.2%) is an efficient alternative if slightly lower accuracy is acceptable. This model enables companies to optimize energy management with accessible data, facilitating the transition to electric fleets without high costs.

A study conducted by Nan et al. [39] addressed the real-time prediction of energy consumption in electric vehicles using a hybrid LSTM-XGBoost model that integrates driving data, road, traffic, and weather information. Using SHAP (Shapley Additive Explanations), the quantitative influence of key variables such as acceleration and temperature was identified. The proposed model demonstrated superiority with an RMSE of 0.079, MAE of 0.086, and R^2^ of 0.814, outperforming traditional approaches. This solution optimizes energy management, reduces operational costs, and mitigates range anxiety in urban electric fleets.

A work by Knox et al. [40] proposed reduced-order hysteretic models and an adaptive extended Kalman filter to accurately estimate the SOC in electric vehicle batteries, where direct measurements are unfeasible. An innovative parameterization framework captures hysteresis effects in NMC811 cells, reducing voltage RMS error by 50% during 18-hour cycles. Integrating the models with the adaptive filter achieves an 85% reduction in SOC estimation error compared to standard industry models.

Our research builds upon the work of Zhang et al. [41], who addressed the challenge of accurately estimating the state of charge in electric vehicle batteries, considering its inherently nonlinear and time-varying nature. Their study systematically reviews SOC estimation techniques described in both scientific literature and patents, classifying them into three main categories: traditional methods (experiment-based), modern methods (rooted in control theory), and emerging intelligent approaches. A particular emphasis is placed on control-based algorithms, especially adaptive and model-based techniques. The authors conclude that control theory remains central to current research, with future developments expected to focus on optimizing data management, improving hardware platforms, refining battery models, and integrating hybrid methodologies to enhance overall system performance.

Although the proposed GA-LSTM model demonstrates high accuracy in estimating the state of charge under dynamic driving conditions and within typical battery operating ranges (10–77% SOC and ambient temperatures between 15–35 °C), its applicability under extreme conditions remains to be fully evaluated. Such conditions include low-temperature environments (e.g., below 0 °C), high C-rate charge/discharge cycles, and operation near voltage cutoffs (SOC levels below 5% or above 95%). Under these scenarios, the battery’s internal impedance and electrochemical behavior may change significantly, leading to nonlinearities and hysteresis phenomena that are not fully captured by the current model. While the proposed method is suitable for most real-world EV applications, future work will address these limitations by expanding the training dataset and enhancing model generalization to ensure robust SOC estimation across a broader range of operating conditions, including thermal extremes and highly dynamic load profiles.

This study contributes to the field by presenting a real-time monitoring system that integrates LSTM neural networks optimized through genetic algorithms and a multiple linear regression model. To contextualize our results, a comparison is made with recent state-of-the-art studies. Previous research has explored a variety of modeling strategies, including LSTM-based hybrid frameworks, regression models enhanced with bio-inspired optimization techniques, and deep learning methods combined with advanced filtering mechanisms. These approaches have demonstrated high predictive accuracy in SOC estimation, though they vary significantly in terms of application scope, data characteristics, and evaluation metrics.

In summary, the findings of this study reinforce the effectiveness of the GA-optimized LSTM model for accurately estimating the state of charge in electric vehicle batteries under dynamic and nonlinear conditions. When benchmarked against multiple linear regression and prior works in the field, the proposed hybrid method consistently outperforms in terms of predictive accuracy, robustness to variable inputs, and adaptability to real-world driving scenarios. Unlike traditional approaches that rely on simplified assumptions or require expert-driven model calibration, the GA-LSTM framework leverages data-driven learning and evolutionary optimization to automatically fine-tune hyperparameters, delivering superior results with minimal manual intervention. While this approach entails higher computational demands, it provides a scalable and adaptable solution for embedded battery management systems with sufficient resources. This work not only validates the model’s capability across various driving cycles but also establishes a methodological foundation for future research aimed at enhancing robustness under extreme conditions. The integration of machine learning with heuristic optimization offers a promising pathway for the development of intelligent, high-performance energy management systems in modern electric vehicles.

Table 8 summarizes key studies in the field, including the authors, methodologies employed, and performance metrics obtained. These investigations highlight the use of numerical modeling, machine learning algorithms, and experimental validations to improve the precision of SOC estimation, driving range prediction, and battery performance under diverse operational conditions in medium- and high-voltage electric vehicles.

## 6. Conclusions

This study has demonstrated that a LSTM network optimized using genetic algorithms is capable of consistently capturing the nonlinear discharge dynamics of electric vehicle batteries under five distinct driving profiles (urban NEDC, extra-urban NEDC, urban WLTP, extra-urban WLTP, and Intercampus). The GA-based optimization process automatically identified the hyperparameter combination that enabled rapid training convergence and the absence of visible overfitting, allowing the model to generalize effectively across highly variable scenarios. Quantitatively, the hybrid LSTM + GA estimator achieved an overall performance of MSE = 0.0426, RMSE = 0.1559, MAE = 0.1299, and R^2^ = 0.9944, confirming its high accuracy and robustness for predicting the state of charge.

The main contribution of this research lies in the application of genetic algorithms for optimal hyperparameter selection in LSTM networks aimed at SOC estimation an innovative approach within the field of electric vehicle mechanics and electronics. By automating and accelerating the search for the most suitable architecture and training parameters, our method paves the way for more accurate and adaptive BMS implementations in real-world environments, where load variability, temperature fluctuations, and cell aging pose constant challenges. Thus, the proposed model not only advances the state of the art in SOC estimation but also sets a precedent for future developments of intelligent battery monitoring and management systems in electric mobility applications.

The multiple linear regression model, despite its simplicity, proved to be a reliable baseline for SOC estimation under varied driving conditions, offering interpretable relationships between input features and state of charge. It achieved an average performance of MSE = 0.9457, RMSE = 0.9725, MAE = 0.8851 and R^2^ = 0.9534, confirming its suitability for applications with limited computational resources or when model transparency is paramount.

The developed monitoring system can adapt to the changing conditions encountered during driving, such as traffic fluctuations, variations in speed, and terrain topography. This adaptability enables real-time adjustments of range predictions, ensuring that they accurately reflect current driving conditions and enhance the precision of the estimates. Additionally, the system can analyze driver behavior, including driving speed, acceleration patterns, and the frequency of stops. Incorporating these data into the range prediction model increases accuracy by considering the impact of driving style on energy consumption.

For future research, addressing the current limitations of the monitoring system offers a strategic foundation for enhancing its functionality and scalability. The integration of emerging technologies and energy optimization strategies will be essential to improve system performance, increase reliability, and reduce operational costs. This multidisciplinary direction supports the sustainable advancement of electric mobility and positions the proposed monitoring system as a critical tool for the efficient management and operation of electric vehicles. For example, the system could leverage navigation and geospatial data to optimize electric vehicle routing and extend driving range. This may include identifying nearby charging stations, planning energy-efficient stopovers, and selecting routes that reduce mechanical resistance and maximize energy recovery. Additionally, the system could generate real-time alerts and notifications when the estimated remaining range reaches critical thresholds, allowing for proactive responses such as rerouting to the nearest charging point or adapting driving behavior to conserve energy.

## Figures and Tables

**Table 1:** Dynamic adjustment of α fusion coefficient.

Condition	Current (A)	α Value
Stable low current	|I|<2 A	0.3 (favor CC)
Moderate current	2≤|I|<8 A	0.5 (equal weight)
High dynamic discharge	|I|≥8 A	0.7 (favor LSTM)

**Table 2:** RMSE between original and filtered signals for different moving average window sizes.

Signal	Window = 3	Window = 5	Window = 7	Window = 9
Raw Voltage	0.1028	0.1346	0.1615	0.1850
Raw Current	1.2025	1.6096	1.9695	2.2522
Raw Acceleration	1.0702	1.3788	1.6596	1.9282

**Table 3:** Signal-to-quantization-noise ratio (SQNR) for different decimal precision levels.

Signal	1 Decimal	2 Decimals	3 Decimals
Raw Voltage	66.37 dB	*∞*	*∞*
Raw Current	54.44 dB	*∞*	*∞*
Raw Acceleration	*∞*	*∞*	*∞*

**Table 4:** Distribution of samples by driving cycle.

Driving Cycle	Number of Samples	Description
NEDC Urban	1043	Low-speed urban cycle, frequent stops
NEDC Extra-Urban	978	Moderate-speed suburban driving
WLTP Urban	1140	Standard urban driving profile
WLTP Extra-Urban	1061	Dynamic acceleration and braking
Inter-Campus Custom Route	991	Mixed-speed route with elevation variation
Total	5213	

**Table 5:** Descriptive statistics of sensor measurements across all driving cycles.

Variable	Mean	Std. Dev.	Min	Max
Voltage (V)	61.98	1.35	58.7	64.1
Current (A)	2.45	5.32	−4.2	26.3
Temperature (°C)	26.1	2.4	22.0	32.7
Acceleration (m/s^2^)	0.88	3.27	−5.7	9.8
Throttle Angle (°)	12.3	8.1	0.0	31.7

**Table 6:** SOC estimator performance metrics (LSTM + GA) in different driving cycles.

Cycle	MSE	RMSE	MAE	$R^{2}$
NEDC urban	0.0054	0.0736	0.0685	0.9987
NEDC extraurban	0.00076	0.0275	0.0232	0.9912
WLTP urbano	0.1554	0.3942	0.3328	0.9925
WLTP extraurban	0.0468	0.2163	0.1637	0.9908
Intercampus	0.0047	0.0682	0.0614	0.9986
Average	0.0426	0.1559	0.1299	0.9944

**Table 7:** Performance comparison between baseline LSTM and GA-optimized LSTM.

Model	MAE (%)	RMSE (%)	Training Time (s)
Baseline LSTM	0.36	0.48	1380
LSTM + GA	0.1299	0.1559	1816

**Table 8:** Compilation of relevant studies.

Author	Applied Methods	Results
(Chandran et al., 2021) [5]	ANN, LR, GPR	ANN: MSE = 0.0004, RMSE = 0.00170; GPR: MSE = 0.023, RMSE = 0.04118.
(Amin et al., 2023) [6]	ML (SVM)	MAE = 0.150.
(Jain et al., 2024) [7]	ML (regressor k-neighbors, model ensemble, Bayesian optimization)	Accuracy: 99.71% (trip 1), 98.04% (trip 2).
(George & Sivraj, 2021) [11]	Bi LSTM DNN	RMSE = 0.029.
(Tian et al., 2021) [13]	DNN integrated with Kalman filter	Error < 2.03%; MSE = 0.385% with significant noise.
(Tiwary et al., 2024) [14]	RNN-LSTM with Adam optimizer	Error < 1% in range estimation for four routes.
(Kumar et al., 2023) [15]	ANN	MAE: 0.0030–0.0035; MSE: 0.0043–0.0047 at 0 °C and 10 °C.
(Gupta & Rahulkar, 2021) [16]	ANN trained with data from FTP-75, HWFET, US06, UDDS cycles	Error < 1% and <3% for WLTP-3 and LA92, respectively.
(Hossain et al., 2023) [18]	Random forest regression (RFR) optimized by DSA	RMSE = 0.382%, MAE = 0.193% (DST) and 0.346% (FUDS).
(Adedeji, 2014) [23]	MANN (Multifunctional Artificial Neural Network)	Case A: MAE = 0.04715, MSE = 0.0064, RMSE = 0.080; Case B: MAE = 0.04719, MSE = 0.007159, RMSE = 0.08461; Case I: MAE = 0.06975, MSE = 0.03618, RMSE = 0.1902.
(Pamuła, 2022) [38]	DLNA, MLR, LSTM	DLNA: RMSE = 0.052, MAPE = 6.2%; MLR: RMSE = 0.055, MAPE = 7.2%; LSTM: RMSE = 0.057, MAPE = 7.2%.
(Nan et al., 2022) [39]	LSTM-XGBoost, MLR	RMSE = 0.079, MAE = 0.086, $R^{2}$ = 0.814.
(Benallal et al., 2025) [42]	LSTM neural networks with Hyperband-based hyperparameter optimization	MSE = 0.0023, MAE = 0.0043.
(Pu et al., 2025) [43]	LSTM and unscented Kalman filter	RMSE = 0.61, MAE = 0.68.
(Li et al., 2023) [44]	Adaboost.Rt-LSTM	MAE = 1.48%, MSE = 2.15%.
(López et al., 2025) [45]	Genetic algorithm-based ESC model	MSE = 2.8, MAE = 28.47.
(Hannan et al., 2021) [46]	Self-supervised transformer model (SSL)	RMSE = 0.90, MAE = 0.40.
(Wei et al., 2020) [47]	NARX-LSTM	RMSE < 1%, 60% improvement over standard LSTM model.
(Y. Chen et al., 2022) [48]	DBC-MLR (Density-based clustering—multiple linear regression)	RMSE of 3.008 kWh/100 km; 11.3%–18.4% improvement over conventional methods.
(Ahmed et al., 2022) [49]	Web mining, correlation analysis (Pearson), SVR	RMSE = 31.4 km, 8.6% accuracy over the average range of 364.5 km.
(Mediouni et al., 2022) [50]	DNN	RMSE = 547.4 W.
(Azkue et al., 2023) [28]	LSTM	MAE = 11.50%.
(How et al., 2020) [51]	DNN with hidden layer configuration	Error < 1% for untrained cycles; 4 optimal hidden layers for accuracy.
(He et al., 2020) [52]	AEKF & RLS	MAE < 0.015 (DST, UDDS), <0.02 with measurement noise.
(Kumari et al., 2023) [53]	ML (analysis of voltage data, internal resistance, SOC)	SOC estimation error: 0.835%.
(G. Zhang et al., 2022) [54]	PSO-RBFNN	MAE = 0.23%, RMSE = 0.34% (NEDC).
(Siva et al., 2022) [55]	NN, SVM (linear kernel), SVM (Gaussian kernel)	NN: RMSE = 0.0079 (training), 0.0019 (validating); SVM-LK: RMSE = 0.1233 (training), 0.0325 (validating); SVM-GK: RMSE = 0.1177 (training), 0.0338 (validating).
(Jagwani et al., 2024) [56]	Linear and logistic regression	More accurate linear regression for SOC on electric motorcycles.
(Bello et al., 2023) [57]	NMPC (non-linear model predictive control)	20% increase in range; 1.5% accuracy in distance traveled estimation.
(Bui et al., 2024) [58]	ML (RRE)	Accuracy: 96.9% (training), 95.8% (testing); 62.6%.
(Xing et al., 2022) [59]	LSTM, BiLSTM, SLSTM, PJTSM	LSTM: RMSE = 2.221; BiLSTM: RMSE = 0.677; SLSTM: RMSE = 2.314; PJTSM: RMSE = 1.836.

## Data Availability

The data supporting the findings of this study are openly available in Mendeley Data at the following link: https://data.mendeley.com/datasets/3vp8cfd69c/1 (accessed on 14 April 2025).

## Abbreviations

The following abbreviations are used in this manuscript:

BDT	Bagged decision tree
BEV	Battery electric vehicle
BMS	Battery management system
CNN	Convolutional neural network
DBC-MLR	Density-based clustering multiple linear regression
EBs	Electric buses
EIS	Impedance spectroscopy
EV	Electric vehicle
GA	Genetic algorithms
GPR	Gaussian process regression
HEVs	Hybrid electric vehicles
HPPC	Hybrid pulse power characterization test
LIBs	Lithium-ion batteries
LSTM	Long short-term memory
MAE	Mean absolute error
MLR	Multiple lineal regression
NEDC	New European Driving Cycle
OCV	Open circuit voltage
PEVs	Pure electric vehicles
PHEV	Plug-in hybrid electric vehicles
PSO	Particle swarm optimization
RFR	Random forest regression
RMSE	Root mean squared error
SVR	Support vector regression
SOC	State of charge
TEC	Total energy consumption
WLTP	WorldwidE Harmonized Light Vehicles Test Procedure
